# The NS2B-PP1α-eIF2α axis: Inhibiting stress granule formation and Boosting Zika virus replication

**DOI:** 10.1371/journal.ppat.1012355

**Published:** 2024-06-27

**Authors:** Xiaoyan Wu, Linliang Zhang, Cong Liu, Qi Cheng, Wen Zhao, Pu Chen, Yali Qin, Mingzhou Chen

**Affiliations:** 1 State Key Laboratory of Virology and Modern Virology Research Center, College of Life Sciences, Wuhan University, Wuhan, China; 2 College of Life Sciences, Hubei University, Wuhan, China; 3 Wuhan Jinyintan Hospital, Tongji Medical College of Huazhong University of Science and Technology, Wuhan, China; 4 Tissue Engineering and Organ Manufacturing (TEOM) lab, Department of Biomedical Engineering, Wuhan University Taikang Medical School (School of Basic Medical Sciences), Wuhan, China; 5 Taikang Center for Life and Medical Sciences, Wuhan University, Wuhan, China; 6 Hubei Jiangxia Laboratory, Wuhan, China; University at Albany State University of New York, UNITED STATES

## Abstract

Stress granules (SGs), formed by untranslated messenger ribonucleoproteins (mRNPs) during cellular stress in eukaryotes, have been linked to flavivirus interference without clear understanding. This study reveals the role of Zika virus (ZIKV) NS2B as a scaffold protein mediating interaction between protein phosphatase 1α (PP1α) and eukaryotic initiation factor 2α (eIF2α). This interaction promotes eIF2α dephosphorylation by PP1α, inhibiting SG formation. The NS2B-PP1α complex exhibits remarkable stability, resisting ubiquitin-induced degradation and amplifying eIF2α dephosphorylation, thus promoting ZIKV replication. In contrast, the NS2B^V35A^ mutant, interacting exclusively with eIF2α, fails to inhibit SG formation, resulting in reduced viral replication and diminished impact on brain organoid growth. These findings reveal PP1α’s dual role in ZIKV infection, inducing interferon production as an antiviral factor and suppressing SG formation as a viral promoter. Moreover, we found that NS2B also serves as a versatile mechanism employed by flaviviruses to counter host antiviral defenses, primarily by broadly inhibiting SG formation. This research advances our comprehension of the complex interplay in flavivirus-host interactions, offering potential for innovative therapeutic strategies against flavivirus infections.

## Author summary

Zika virus (ZIKV) belongs to vector-borne flaviviruses, associated with Guillain-Barré syndrome and neonatal microcephaly. Stress granules (SGs) which forms under stress in eukaryotic cells can interfere with viral replication. Although previous studies have indicated that ZIKV can interfere with SG formation, the precise molecular mechanism and function remain unclear. Exploration of the mechanisms by which ZIKV antagonizes SG formation may help to identify new antiviral approaches. Here, we found that ZIKV NS2B bridge PP1α and eIF2α to promote eIF2α dephosphorylation and SG inhibition. Meanwhile, Reciprocal inhibition of K48-linked ubiquitination stabilized NS2B and PP1α to further facilitate the dephosphorylation of eIF2α and the concurrent inhibition of SG formation. Conversely, we demonstrated that a mutant form of NS2B, NS2B^V35A^, which interacted solely with eIF2α and not PP1α, attenuated virus replication and mitigated neurotoxicity in brain organoid development. The NS2B-PP1α-eIF2α axis reveals a novel biological function of NS2B as a general mechanism for flavivirus evasion of host antiviral effects, providing new therapeutic strategies for antiviral study.

## Introduction

Stress granules (SGs) represent cytoplasmic assemblages of messenger ribonucleoproteins (mRNPs) characterized by a dynamic nature, comprising a condensed core and a less compact periphery [1]. Recent investigations have shown the antiviral attributes of SGs, and mounting evidence emphasizes the potential for viral elements to disrupt SG assembly [2,3]. The process of SG formation involves multiple aspects, including various protein modifications at key sites and interactions involving RNA-binding proteins and the cytoskeleton [3,4]. Viruses can modify many crucial steps in the formation of SGs to facilitate their replication [5].

The Flavivirus genus encompasses more than 70 viral species, some of which are notable human pathogens, such as Dengue virus (DENV), Yellow fever virus (YFV), West Nile virus (WNV), Japanese encephalitis virus (JEV) and Zika virus (ZIKV) [6]. The Flavivirus genome consists of a positive-sense, single-stranded RNA molecule approximately 11,000 nucleotides in length. It is characterized by a 5’-untranslated region (UTR) with a standardized cap structure, a single open reading frame (ORF), and a 3’-UTR that, in contrast to polyadenylation (with the exception of TBEV), forms complex RNA structures serving equivalent functions. The ORF encodes a polyprotein that undergoes co-translational processing by both viral and host proteases, resulting in ten mature viral proteins: three structural (C, prM/M and E) and seven nonstructural (NS) proteins (NS1, NS2A, NS2B, NS3, NS4A, NS4B and NS5) [7]. NS2B, a transmembrane protein located within the endoplasmic reticulum (ER), predominantly serves as a cofactor for NS3 [8]. NS2B interacts with NS3, providing structural stabilization, acting as an ER anchoring point, and enabling the activation of NS3’s enzymatic region, thereby conferring protease activity [9]. Nevertheless, limited evidence has been available to support an independent biological role for NS2B. Recent studies have shed light on the various functions of NS2B. For instance, ZIKV NS2B can impede TBK1 phosphorylation, consequently inhibiting IFN-β production [10]. DENV NS2B has been observed to directly interact with cGAS, facilitating its degradation via autophagy-lysosome processes and subsequently diminishing STING-mediated interferon production [11]. Moreover, DENV NS2B has been implicated in the targeting of MAVS and IKKε, thereby weakening the RIG-I-directed antiviral response [12]. The cytoplasmic region spanning amino acids 51 to 95 of NS2B is adequate for sustaining NS3 protease activity, while the precise role of the transmembrane domain (TMD) remains unclear. Additionally, JEV NS2B plays a critical role in virus particle assembly [13].

The Protein Phosphatase 1C (PPP1C or PP1c) family encompasses three serine/threonine protein phosphatase subtypes: PPP1CA (PP1α), PPP1CB (PP1β), and PPP1CC (PP1γ), characterized by similar substrate specificity [14]. PPP1c plays an important role in regulating various cellular antiviral defenses. Their primary functions include glycogen metabolism, gene transcription and cell cycle progression [15–17]. Especially, the dephosphorylation of key antiviral molecules such as MDA5 and RIG-I by PP1α and PP1γ serves to activate IFNβ [18]. PDEV nsp7 attenuates MDA5-PP1α/-γ interactions, thereby effectively suppressing MDA5 dephosphorylation and its subsequent activation [19]. HIV Tat engages in interactions with PP1γ, orchestrating the dephosphorylation of CDK9 and activating HIV-1 transcription [20]. Furthermore, ASFV DP71L [21] and TGEV protein 7 [22] bind to PP1c and facilitate the recruitment of PP1c for the dephosphorylation of eIF2α.

Current research endeavors aimed at inhibiting SGs during flavivirus infection primarily revolves around two main strategies: attenuation of eIF2α phosphorylation and hijacking the core SG markers. However, the precise molecular mechanisms underlying these strategies remain unclear. To bridge this knowledge gap, we explored ZIKV infection. On one hand, ZIKV disrupts SG assembly through the utilization of specific viral proteins such as NS3, NS4A, and capsid proteins. The capsid protein interferes with SG formation by interacting with G3BP1 and Caprin-1, facilitating viral replication [23]. ZIKV strategically manipulates core SG proteins—G3BP1, TIAR, and Caprin-1—disrupting SG assembly and repurposing them for its own replication [24]. On the other hand, ZIKV attenuates eIF2α phosphorylation to hinder SG formation [25,26]. Earlier investigations focus on describing the phenomenon of SG inhibition without a comprehensive molecular mechanism, especiallly regarding the specific mechanisms of inhibiting eIF2α phosphorylation. Exploring the potential to disrupt ZIKV replication by targeting SG formation or their interactions with viral components could lead to innovative antiviral strategies. Additionally, ZIKV has been associated with triggering neurological complications like microcephaly [27], while SGs have links to neurodegenerative diseases [28,29]. Understanding the influence of disrupted SG assembly on neural development is important for comprehending its pathogenesis. Given the key role of SG assembly in ZIKV replication, a pressing question arises: what precise molecular mechanisms underlie the disruption of SG assembly by ZIKV?

Our investigations demonstrate that ZIKV NS2B effectively inhibits the formation of drug-induced SGs by orchestrating the recruitment of PP1α, which subsequently dephosphorylates eIF2α. Significantly, NS2B serves as the molecular bridge linking PP1α and eIF2α to catalyze eIF2α dephosphorylation, entirely independent of GADD34. Furthermore, our investigation reveals a mutual stabilization between NS2B and PP1α. This stability stems from their collective ability to inhibit K48-linked ubiquitination. Additionally, our research reveals a dual role played by PP1α in ZIKV replication, involving the inhibition of SG formation on one hand, and the induction of IFN production on the other. Collectively, our study is the first to elucidate that ZIKV NS2B utilizes the NS2B-PP1α-eIF2α axis to block SG formation, thus promoting virus replication. This mechanism emerges as a unifying strategy among flavivirus NS2B proteins.

## Results

### Inhibition of SG formation by ZIKV NS2B through eIF2α dephosphorylation

**Fig 1:**
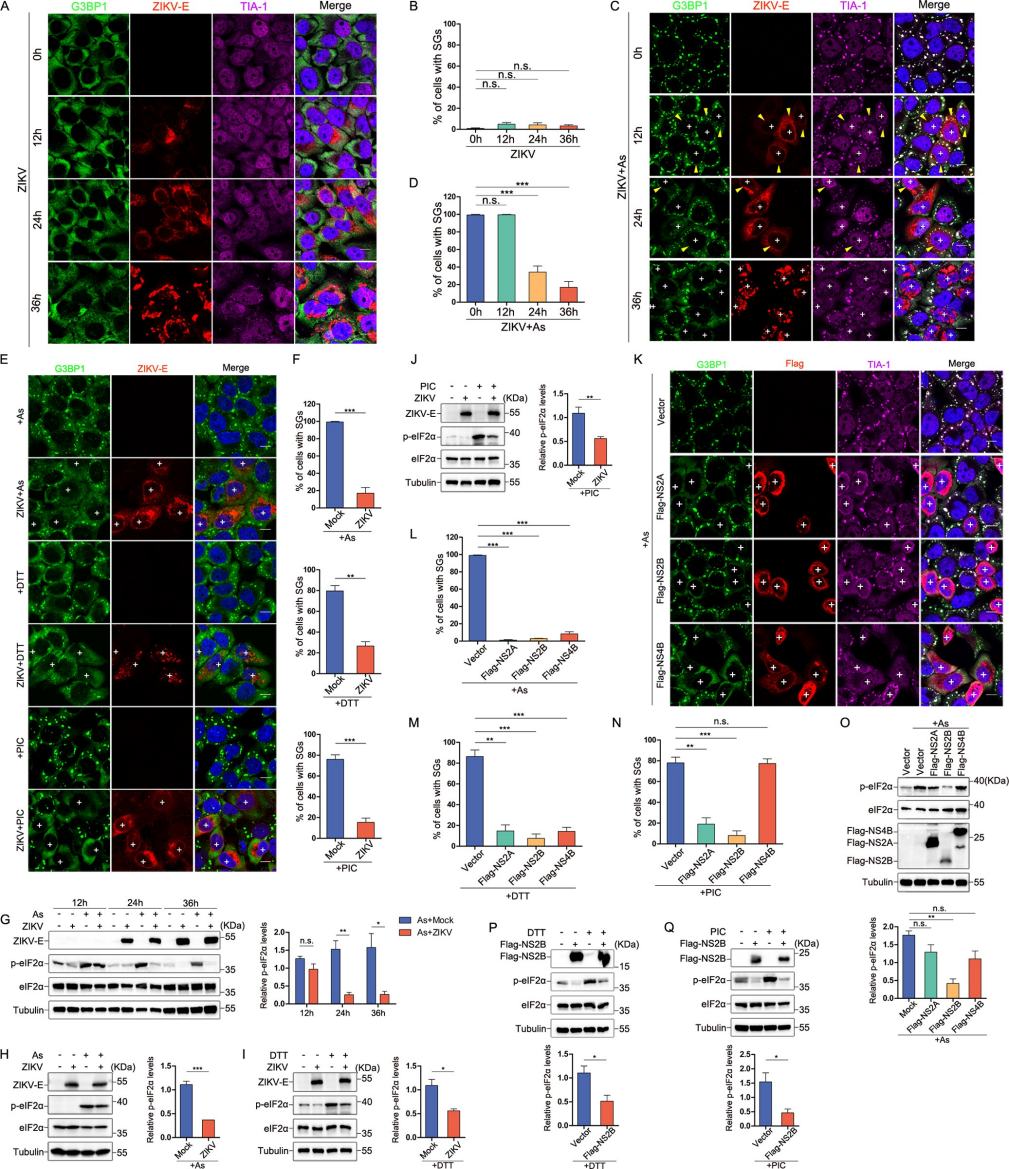
NS2B-mediated eIF2α dephosphorylation suppresses SG formation during ZIKV infection. **(A)** HeLa cells were infected with ZIKV (MOI = 0.3) at the indicated time points, Immunostaining for G3BP1 (green) ZIKV-E (red) and TIA-1 (magenta) was conducted thereafter. **(B)** The percentage of cells containing SGs was determined by analyzing images obtained from panel (A) in three independent experiments. **(C)** HeLa cells were infected with ZIKV (MOI = 0.3) at the indicated time points, before being harvested treated with As (200 μM) for 1hour. Immunostaining for G3BP1 (green) ZIKV-E (red) and TIA-1 (magenta) was conducted thereafter. **(D)** The percentage of cells containing SGs was determined by analyzing images obtained from panel (C) in three independent experiments. **(E)** HeLa cells were either mock-infected or infected with ZIKV (MOI = 0.3) for 36 hours, before being harvesting treated with As (200 μM) for 1hour, DTT (2 nM) for 1hour, or PIC (2 ng) for 12hours. Immunostaining for G3BP1 (green) and ZIKV-E (red) was conducted thereafter. **(F)** The percentage of cells containing SGs was determined by analyzing images obtained from panel (E) in three independent experiments. **(G)** HeLa cells were infected with ZIKV (MOI = 0.3) at the indicated time points, before being harvested untreated or treated with As (200 μM) for 1hour. Following treatment, cell lysates were analyzed via western blot using antibodies specific to phosphorylated eIF2α, eIF2α, ZIKV-E and tubulin. Also shown is the intensity quantification of p-eIF2α at 12, 24, 36 hours following As treatment, as determined by ImageJ analysis. The values presented in the graph are the relative intensity ratio of p-eIF2α to eIF2α. **(H to J)** HeLa cells were either mock-infected or infected with ZIKV for 36hours, before being harvested untreated or treated with As (200μM) for 1hour (H), DTT (2nM) for 1hour (I), or PIC (2ng) for 12hours (J). Following treatment, cell lysates were analyzed via Western blot using antibodies specific to phosphorylated eIF2α, eIF2α, ZIKV-E, and tubulin. Also shown is the intensity quantification of p-eIF2α when treated with As (H), DTT (I) or PIC (J), as determined by ImageJ analysis. Values presented in the graph are the relative intensity ratio of p-eIF2α to eIF2α. **(K and L)** Hela cells were transfected with either empty vector or plasmids expressing Flag-tagged NS2A, NS2B, or NS4B for 24 hours, followed by As (200μM) treatment for 1hour. (K) Immunofluorescence analysis was conducted utilizing antibodies against G3BP1 (in green), HA (in red), and TIA-1 (in magenta). (L)The percentage of cells containing SGs from panel K was quantified across three independent experiments. **(M and N)** The quantification of cells containing SGs was carried out on HeLa cells transfected with either an empty vector, Flag-NS2A, Flag-NS2B, or Flag-NS4B plasmids for 24 hours, followed by treatment with DTT (2nM) for 1hour (M) or PIC (2ng) for 12hours (N). The results represent data obtained from three independent experiments. **(O)** HeLa cells were transfected with empty vector or plasmids expressing Flag-NS2A, Flag-NS2B, or Flag-NS4B for 24hours, and subsequently treated with As (200 μM) for 1hour. Cell lysates were then analyzed via Western blot, using anti-phosphorylated eIF2α, anti-eIF2α, anti-Flag, and anti-tubulin antibodies. Also shown is intensity quantification of p-eIF2α when treated with As, as determined by ImageJ analysis. Values presented in the graph are the relative intensity ratio of p-eIF2α to eIF2α. **(P and Q)** HeLa cells were transfected with empty vector or plasmids expressing Flag-NS2B for 24 hours, and subsequently untreated or treated with DTT (2nM) for 1hour (P) or PIC (2ng) for 12hours (Q). Cell lysates were then analyzed via Western blot, using anti-phosphorylated eIF2α, anti-eIF2α, anti-Flag, and anti-tubulin antibodies. Also shown is intensity quantification of p-eIF2α when treated with DTT (P) or PIC (Q), as determined by ImageJ analysis. Values presented in the graph are the relative intensity ratio of p-eIF2α to eIF2α. Cell with a particle count of at least 3 and a particle diameter within the range of 1.5–2 μm as SG-positive cell. The symbol ’+’ denotes cells that exhibited inhibition of SG formation, while cells marked with both "+" and yellow arrowheads indicate those that did not. The white scale bar represents a length of 10μm. Error bars indicate the standard deviation (SD) with a sample size (n) of 3. In each experiment, a total of 150 cells were counted. Statistical significance was determined through a Student’s t-test, where ’n.s.’ indicates no significance, *P < 0.05, **P < 0.01, and ***P < 0.001.

To elucidate the molecular complexities governing SG formation during ZIKV infection, we conducted a dynamic time course analysis of SG formation in Hela cells infected with or without arsenite (As) treatment. Results showed that ZIKV not only failed to induce SG formation (Fig 1A and 1B) but also inhibited SG formation induced by As, with the inhibition rate of 80% at 36 hours post-infection (Fig 1C and 1D). We also performed infection experiments on HeLa cells for 36hours and investigated SG formation using immunofluorescence (IF) assays with specific antibodies against other SG markers, including eIF4E, eIF4G1, and PABP1. The results were consistent with our previous findings, confirming the presence of G3BP1 foci as likely bona fide SGs (S1A–S1D Fig). To exclude cell-specific effects, we infected A549 cells with ZIKV for 24hours, treated with or without As. Similar results were observed in ZIKV-infected A549 cells (S1E–S1H Fig). Additionally, we explored the inhibitory effects of ZIKV on SGs, using three drugs to induce SG formation, demonstrating that ZIKV can inhibit SGs induced by As, dithiothreitol (DTT) and poly(I:C) (PIC) (Fig 1E and 1F).

Furthermore, our investigation demonstrated several key observations. Initially, ZIKV infection was found to activate PKR (S2A Fig), a notable event coinciding with the virus’s suppression of eIF2α phosphorylation in response to As, DTT, and PIC (Fig 1G–1J). This suggests that ZIKV employs a strategy of inhibiting SG formation by inducing the dephosphorylation of eIF2α. In seeking to identifying the viral element(s) responsible for this SG inhibition, we expressed each viral protein in HeLa cells and examined their impact on SG formation induced by As, DTT, and PIC. Our results revealed that NS2A, NS2B, and NS4B demonstrated a shared ability to block SG formation in response to As and DTT (Fig 1K–1M). Notably, NS2A and NS2B effectively inhibited SG formation induced by PIC (Fig 1N). NS2B significantly attenuated the phosphorylation of eIF2α upon As exposure (Fig 1O). Additionally, NS2B demonstrated inhibition of eIF2α phosphorylation induced by DTT (Fig 1P) and PIC (Fig 1Q), indicating that NS2B inhibits SG formation by specifically targeting the eIF2α signaling pathway.

### NS2B mediates eIF2α dephosphorylation via PP1α

**Fig 2:**
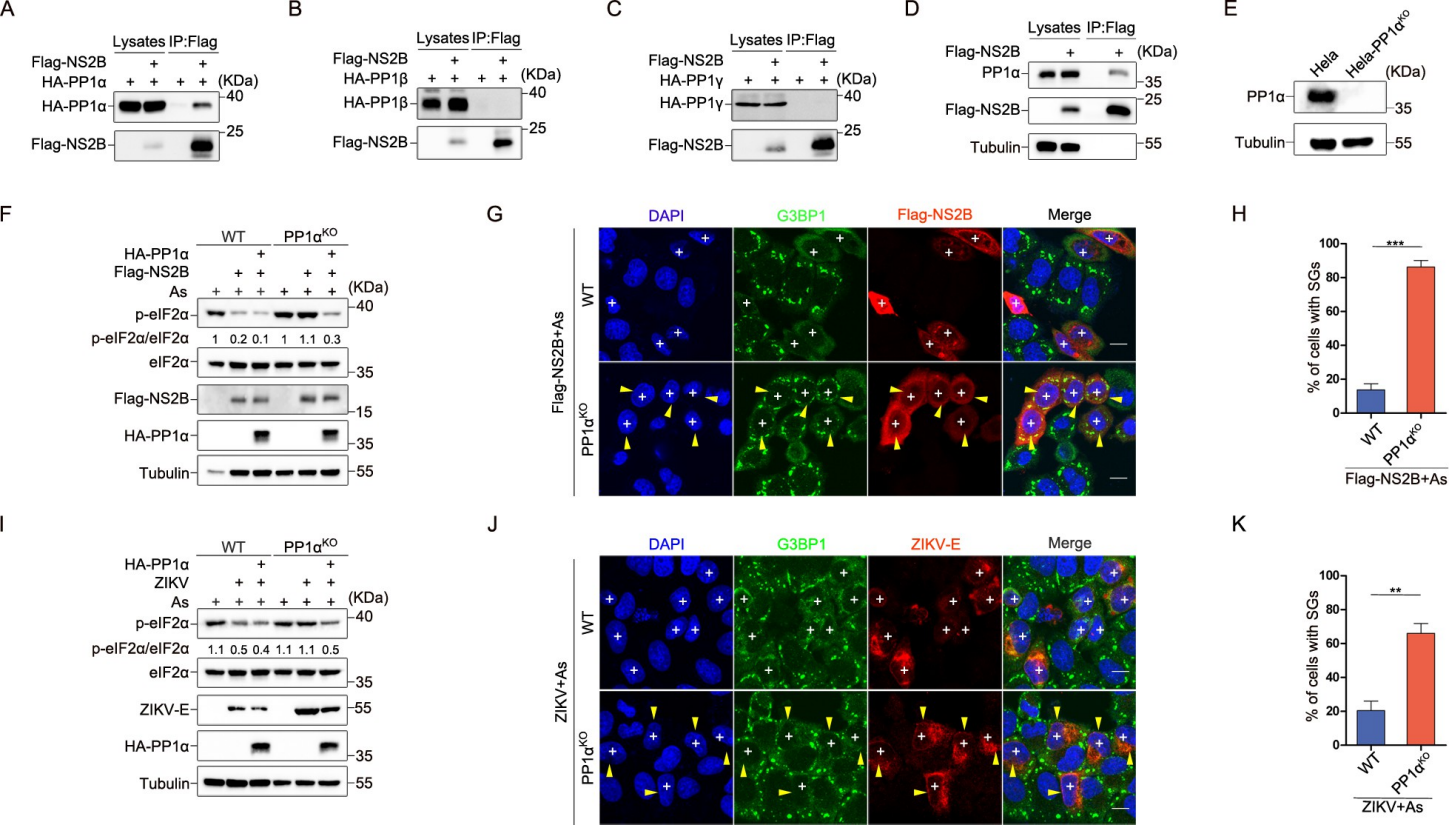
NS2B facilitates eIF2α dephosphorylation through PP1α. **(A to C)** HEK293T cells transfected with empty vector or Flag-NS2B, co-transfected with HA-PP1α (A), HA-PP1β (B), or HA-PP1γ (C) for 24hours, followed by IP of protein lysates using anti-FLAG affinity Gel. **(D)** HEK293T cells transfected with empty vector or Flag-NS2B for 24hours, followed by IP of protein lysates using anti-FLAG affinity Gel. **(E)** Western blot analysis of PP1α in Hela wild-Type (WT) cells and Hela-PP1α^KO^ cell clones. **(F)** Empty vector, Flag-NS2B, and Flag-NS2B along with HA-PP1α carrying synonymous mutations were transfected into both Hela-WT and Hela-PP1α^KO^ cells for 24hours. Afterward, the cells were treated with As (200 μM) for 1hour, and p-eIF2α/eIF2α quantification was performed following immunostaining. **(G and H)** Flag-NS2B was introduced into Hela-WT or Hela-PP1α KO cells for 24hours, and these cells were subsequently treated with As (200 μM) for 1hour. The experiment involved immunostaining (G) and quantification of SGs (H). **(I)** ZIKV-infected Hela-WT or Hela-PP1α^KO^ cells were transfected with HA-PP1α containing synonymous mutations for 36hours, followed by As (200 μM) treatment for 1hour before being harvested. Protein lysates were then subjected to Western blot analysis. **(J and K)** ZIKV infected Hela-WT and Hela-PP1α KO cells for 36hours, followed by As (200 μM) treatment for 1hour before being harvested. Cells were subjected to immunostaining (J) and quantification of SGs (K). Cells labeled with "+" indicate NS2B-expressing or ZIKV-infected cells that inhibited SG formation, while cells marked with both "+" and yellow arrowheads indicate those that did not. A 10 μm white scale bar is provided, and error bars represent the standard deviation of three independent experiments, with 150 cells counted each time. Statistical significance was assessed using the Student’s t-test, with "n.s." indicating no significance, *P < 0.05 denoting significance at the 5% level, **P < 0.01 at the 1% level, and ***P < 0.001 at the 0.1% level.

Subsequently, we investigated the precise mechanism through which NS2B dephosphorylated eIF2α. We employed immunoprecipitation-mass spectrometry (IP-MS) to identify NS2B-associated proteins. Two phosphatases, PP1α and PP1γ, were considered valuable interacters interacting with NS2B. Further analysis revealed that NS2B exhibited a specific affinity for PP1α, distinguishing it from PP1β and PP1γ, as evidenced by co-immunoprecipitation (Fig 2A–2D) and co-localization (S3A Fig) studies. To validate the hypothesis that NS2B’s dephosphorylation of eIF2α rely on PP1α, we generated HeLa-PP1α knockout (KO) cells (Fig 2E) and HEK293T-PP1α knockout (KO) cells (S3B Fig). These experiments revealed a significant reduction in eIF2α dephosphorylation by NS2B in the absence of PP1α, a deficiency that was effectively restored upon exogenous replenishment of PP1α (Figs 2F and S3C). Moreover, our research showed that the ability of NS2B to hinder SG formation was significantly impaired in HeLa-PP1α KO cells compared to their wild-type counterparts (Fig 2G and 2H). Similarly, we observed a significant impairment in eIF2α dephosphorylation (Fig 2I) and SG formation inhibition (Fig 2J and 2K) in ZIKV-infected HeLa-PP1α KO cells. These results demonstrated that ZIKV’s NS2B mediated eIF2α dephosphorylation to inhibit SG formation via PP1α. Additionally, we identified a non-functional NS2B single amino acid substitution (V35A) through a stepwise construction of truncation and point mutants. We found that NS2B^ΔN1-34^ retained their ability to inhibit SG formation, while NS2B^ΔN1-36^ did not, indicating the importance of residues 35 and 36. Consequently, we performed mutations including V35A, G36A, and VG3536AA, confirming that V35 is the critical site responsible for this functional change (S3D Fig). This mutant exhibited an inability to suppress SG formation (S3E and S3F Fig) and dephosphorylate eIF2α (S3G Fig). Interestingly, NS2B^V35A^ also displayed a substantial reduction in its interaction with PP1α when compared to the wild-type NS2B, underscoring the pivotal role of the NS2B-PP1α interaction in eIF2α dephosphorylation and SG formation inhibition (S3H Fig).

### NS2B serves as a bridge between PP1α and eIF2α for eIF2α dephosphorylation

**Fig 3:**
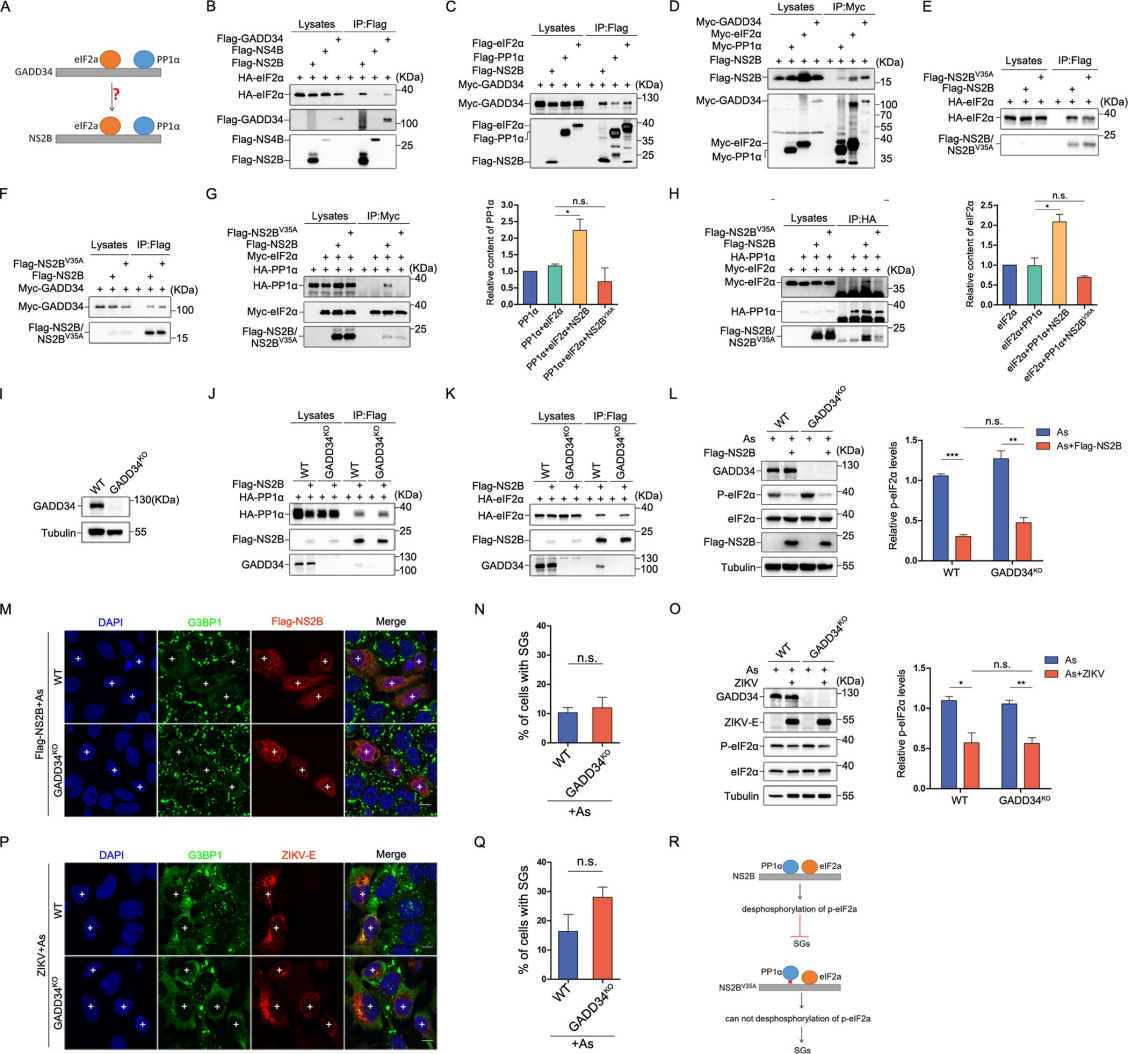
NS2B facilitates eIF2α dephosphorylation independently of GADD34. **(A)** A schematic diagram shows NS2B bridging PP1α and eIF2α to promote eIF2α dephosphorylation. **(B)** In HEK293T cells, immunoprecipitation assays were conducted to investigate the interactions among Flag-tagged NS2B, NS4B, GADD34 or the empty vector control and HA-tagged eIF2α. **(C)** In HEK293T cells, immunoprecipitation assays were conducted to assess the interactions between Flag-tagged NS2B, PP1α, eIF2α or the empty vector control and Myc-tagged GADD34. **(D)** In HEK293T cells, immunoprecipitation assays were conducted to investigate the interactions among Myc-tagged PP1α, eIF2α, GADD34, or the empty vector control, and Flag-tagged NS2B. **(E and F)** Immunoprecipitation assays were performed to assess the interaction of NS2B, NS2B^V35A^ with HA-tagged eIF2α (E), or Myc-tagged GADD34 (F) in HEK293T cells. **(G and H)** Immunoprecipitation assays were performed to explore the impact of NS2B or NS2B^V35A^ on the interplay between eIF2α and PP1α. Also shown is quantification analysis of the PP1α (G) or eIF2α (H) content in co-IP. **(I)** Western blot analysis was employed to identify the presence of GADD34 in both Hela WT cells and Hela-GADD34^KO^ cell clones. **(J and K)** In HEK293T cells, immunoprecipitation assays were conducted to investigate the influence of GADD34 on the interaction between Flag-tagged NS2B and HA-tagged PP1α (J) or eIF2α (K). **(L)** Western blot analysis was carried out to assess the impact of GADD34 KO on NS2B-mediated eIF2α dephosphorylation in Hela cells. **(M and N)** Immunofluorescence analysis (M) and subsequent quantification of SGs (N) were undertaken to explore the influence of GADD34 KO on NS2B-mediated inhibition of SG formation in Hela cells. **(O)** Western blot analysis was performed to investigate the impact of GADD34 KO on ZIKV-mediated eIF2α dephosphorylation in Hela cells. **(P and Q)** Immunofluorescence analysis (P) was followed by quantification of SGs (Q) to explore the impact of GADD34 KO on ZIKV-mediated inhibition of SG formation in Hela cells. **(R)** NS2B bridges PP1α and eIF2α, promoting eIF2α dephosphorylation and suppressing SG formation, while this function is absent in NS2B^V35A^. Cells labeled with "+" indicate NS2B-expressing or ZIKV-infected cells that inhibited SG formation. A 10 μm white scale bar is provided, and error bars represent the standard deviation of three independent experiments, with 150 cells counted each time. Statistical significance was determined by Student’s t-test: n.s. (not significant), *P < 0.05, **P < 0.01, ***P < 0.001. Scale bar: 10μm.

Subsequently, our focus shifted to the investigation of the mechanisms governing NS2B-mediated eIF2α dephosphorylation through PP1α. Previous studies have highlighted the role of GADD34 as a bridging factor between PP1α and eIF2α for eIF2α dephosphorylation [30]. Additionally, various other proteins, such as the DP71L protein of ASFV [21], protein 7 of TGEV (22) and ICP34.5 protein of HSV-1 [31] have been identified as specific facilitators of eIF2α dephosphorylation through their bridging interaction with PP1α and eIF2α. We hypothesized that NS2B might function in a similar way, dephosphorylating eIF2α by bridging PP1α and eIF2α (Fig 3A). Our initial result confirmed the interaction between NS2B and PP1α (Fig 2A and 2D), and demonstrated that the functionally impaired NS2B^V35A^ mutant loses its ability to interact with PP1α (S3H Fig). Subsequently, we sought to verify the interactions between NS2B and the NS2B^V35A^ mutant with eIF2α. Our findings confirmed that both NS2B (Fig 3B–3D) and NS2B^V35A^ (Fig 3E and 3F) interact with eIF2α as well as with GADD34, as demonstrated by immunoprecipitation. Meanwhile, we observed that NS2B exhibited specific co-localization with PP1α and eIF2α, while NS2B^V35A^ did not co-localize with PP1α (S4A and S4B Fig). Notably, NS2B was found to enhance the interaction between PP1α and eIF2α, a capacity absent in NS2B^V35A^ (Fig 3G and 3H). These results indicated that NS2B may function as a bridge linking PP1α and eIF2α. However, given that NS2B also interacts with GADD34, we cannot exclude the possibility that this bridging interaction may be mediated by GADD34. To study deeper into this mechanism, we generated HeLa-GADD34 knockout (KO) cells (Fig 3I), and found that the interaction (Fig 3J and 3K) and colocalization (S4C and S4D Fig) of NS2B with PP1α and eIF2α remained unaffected in these cells, highlighting the independence of NS2B’s interaction with PP1α and eIF2α from GADD34. Furthermore, we observed no significant difference in NS2B’s capacity to dephosphorylate eIF2α (Fig 3L) or inhibit SG formation (Fig 3M and 3N) between Hela WT and Hela-GADD34 KO cells. Similarly, there was no significant difference in the ability of ZIKV to dephosphorylate eIF2α (Fig 3O) or inhibit SG formation (Fig 3P and 3Q) between Hela WT and Hela-GADD34 KO cells. These collective findings affirm the role of NS2B as a bridge linking PP1α and eIF2α, thereby facilitating eIF2α dephosphorylation, independently of GADD34 (Fig 3R).

### Reciprocal inhibition of K48-linked ubiquitination stabilizes NS2B and PP1α

**Fig 4:**
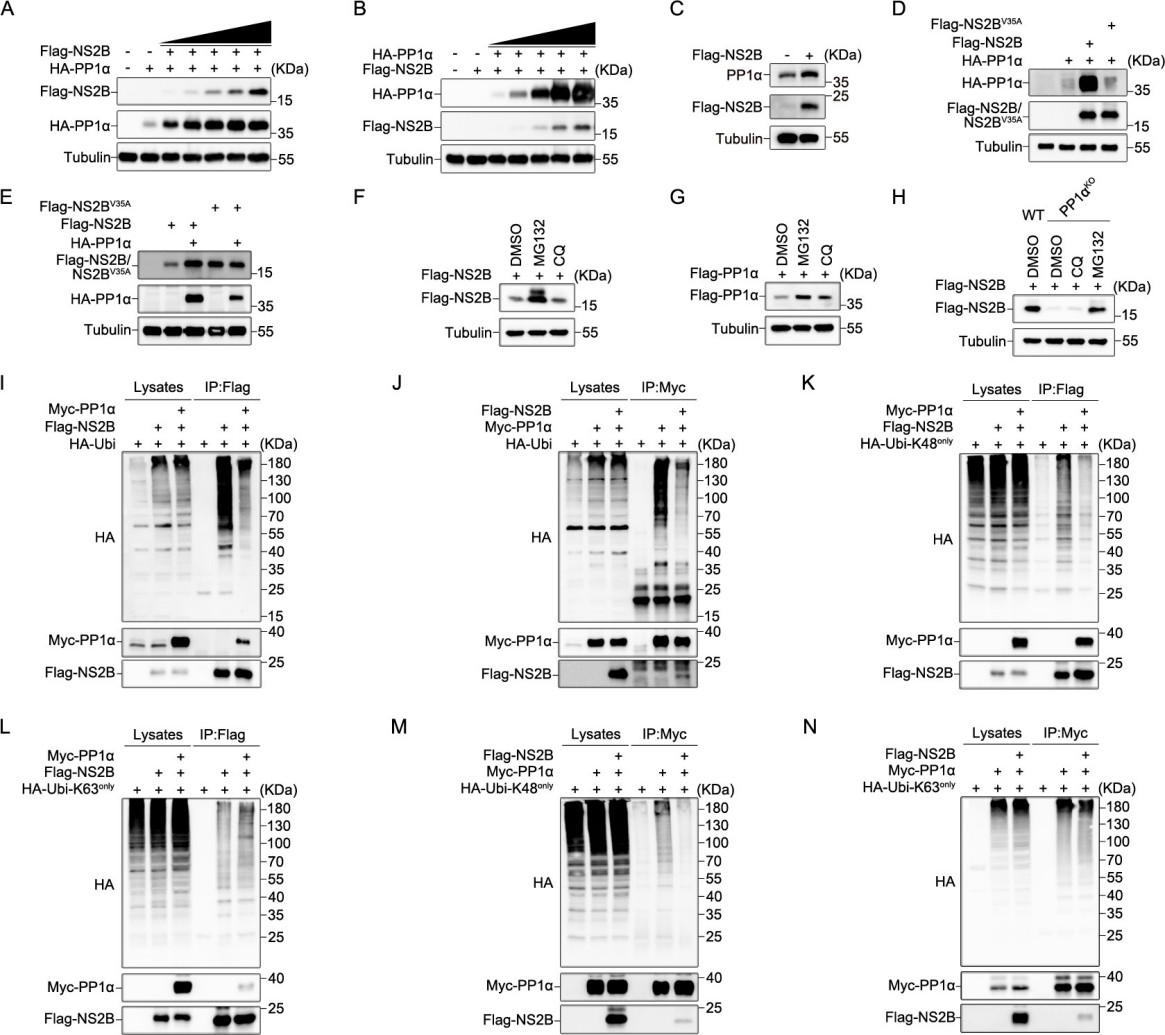
Reciprocal K48-linked ubiquitination inhibition enhances the stability of NS2B and PP1α. **(A)** The impact of Flag-NS2B on HA-PP1α expression was assessed by transfecting 0.2μg HA-PP1α into HEK293T cells with increasing concentrations of Flag-NS2B and corresponding concentration supplementary empty vector. **(B)** The impact of HA-PP1α on Flag-NS2B expression was assessed by transfecting 0.5μg Flag-NS2B into HEK293T cells with increasing concentrations of HA-PP1α and corresponding concentration supplementary empty vector. **(C)** HEK293T cells were transfected with empty vector or Flag-NS2B plasmids to assess their impact on endogenous PP1α expression. **(D and E)** The reciprocal effects of NS2B^V35A^ and PP1α expression on each other’s protein levels were examined. **(F and G)** The degradation pathways of NS2B and PP1α were explored by transiently transfecting HEK293T cells with Flag-NS2B (F) or Flag-PP1α (G) and subjecting them to treatment with 20mM MG132 for 8hours or 50μM CQ for 6 hours before being harvested. **(H)** HEK293T-WT and HEK293T-PP1α^KO^ cell lines were transfected with Flag-NS2B and exposed to 20mM MG132 for 8hours or 50μM CQ for 6 hours before being harvested. **(I, K and L)** The role of PP1α in NS2B ubiquitination was investigated in HEK293T cells, with a focus on polyubiquitination, K48-linked ubiquitination, and K63-linked ubiquitination. **(J, M and N)** The impact of NS2B on PP1α ubiquitination was examined in HEK293T cells, specifically in polyubiquitination, K48-linked ubiquitination, and K63-linked ubiquitination.

To investigate how the interaction between NS2B and PP1α orchestrates eIF2α dephosphorylation and the consequent inhibition of SG formation, we conducted a detailed analysis of their mutual effects. We observed a reciprocal enhancement in the expression of NS2B and PP1α by one another (Fig 4A and 4B). Meanwhile, we observed that NS2B can upregulate the expression of endogenous PP1α (Fig 4C). However, this enhancement was absent in the case of NS2B^V35A^ and PP1α (Fig 4D and 4E). Nest, by using proteasome inhibitor MG-132 and autophagosome inhibitor chloroquine (CQ), our examination further found that both NS2B and PP1α were subject to ubiquitination-mediated degradation processes (Fig 4F and 4G). In HEK293T-PP1α KO cells, the expression of NS2B experienced a substantial reduction. However, this decrease was promptly reversed by treatment with the proteasome inhibitor MG-132 (Fig 4H), and the expression of PP1α significantly inhibited the polyubiquitination of NS2B (Fig 4I). This indicated that PP1α plays an important role in hindering the proteasomal degradation of NS2B. Similarly, NS2B exhibited the ability to inhibit the polyubiquitination of PP1α (Fig 4J). It is important to note that transient transfection of mammalian cells with plasmids induces stress-induced translation arrest through PKR. Therefore, we cannot ignore the contribution of NS2B-eIF2α dephosphorylation to the upregulation of PP1α and NS2B expression. Additionally, our research also extended to the specific ubiquitination mechanisms underlying NS2B and PP1α. We conducted experiments involving the expression of HA-K48-Ubi and HA-K63-Ubi. Our findings demonstrated that PP1α significantly reduced the K48-linked ubiquitination level of NS2B, with only a slight increase in the K63-linked ubiquitination level of NS2B (Fig 4K and 4L), NS2B reduced the K48 ubiquitination level of PP1α, while having minimal effect on K63 ubiquitination (Fig 4M and 4N). These collective findings underscore the reciprocity in which NS2B and PP1α inhibit the K48-linked ubiquitination processes, thereby stabilizing their expression. This stability, in turn, facilitates the dephosphorylation of eIF2α and the consequent inhibition of SG formation.

### Dual role of PP1α in ZIKA Infection: antiviral and proviral functions

**Fig 5:**
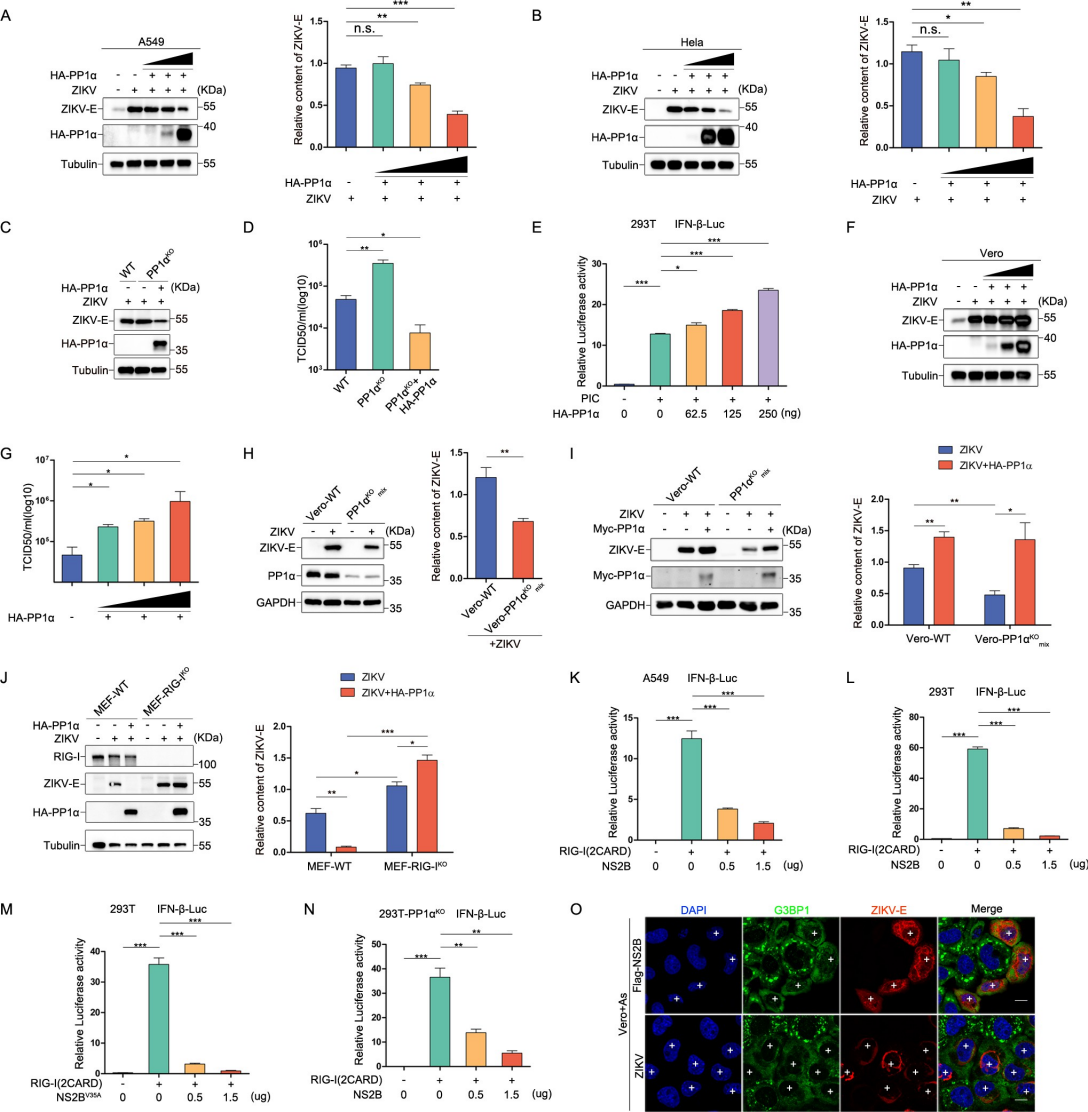
PP1α exhibits a dual role during ZIKV infection. **(A and B)** The impact of HA-PP1α on ZIKV replication was assessed by transfecting escalating concentrations of HA-PP1α and corresponding concentration supplementary empty vector into A549 cells (A) or Hela cells (B), followed by ZIKV infection for 36 hours after a 12-hour transfection period. Cell lysates were analyzed via western blot. Also shown is quantification analysis of the ZIKV-E content. **(C and D)** Hela-WT and Hela-PP1α^KO^ cells were infected with ZIKV (MOI = 0.3) and transfected with HA-PP1α containing synonymous mutations and corresponding concentration supplementary empty vector for 36 hours. (C) Cell lysates were analyzed via Western blot, and (D) Cell supernatants were then utilized to determine TCID50. **(E)** The impact of PP1α on the IFN reporter system was assessed in HEK293T cells. Cells were transfected with either an empty vector or HA-PP1α, in combination with IFN-β luciferase reporter (IFN-β-Luc, 200 ng), PRL-TK (20 ng), and PIC (0.25 μg) for a duration of 36 hours prior to harvesting. Subsequently, dual luciferase reporter assays were executed. **(F and G)** The influence of PP1α on ZIKV replication in Vero cells lacking an interferon system was examined by transfecting escalating doses of HA-PP1α for a 12-hour period, followed by ZIKV infection for 36 hours. Cell lysates were subsequently subjected to Western blot analysis (F), while cell supernatants were employed to determine TCID50 (G). **(H and I)** Further evaluation of the impact of PP1α on ZIKV replication in Vero cells was conducted. In (H), both Vero-WT and Vero-PP1α^KO^ mix cells were either mock-infected or infected with ZIKV. Subsequently, in (I), Vero-WT and Vero-PP1α KO mix cell lines were subjected to mock infection, ZIKV infection, or ZIKV infection followed by transfection with Myc-PP1α carrying synonymous mutations for 36 hours. Cell lysates were then analyzed via western blot. Also shown is quantification analysis of the ZIKV-E content in ZIKV-infected cells. **(J)** ZIKV infected MEF-RIG-I^WT^ and MEF-RIG-I^KO^ cells, were transfected with HA-PP1α for 36hours. Protein lysates were then subjected to western blot analysis. Also shown is quantification analysis of the ZIKV-E content in ZIKV-infected cells. **(K and L)** Assessing NS2B’s Impact on the IFN Reporter System: A549 cells (K) and HEK293T cells (L) were transfected with either empty vector or Flag-NS2B, along with IFN-β luciferase reporter (IFN-β-Luc, 200 ng), PRL-TK (20 ng), and HA-RIG-I-(2CARD) (0.25 μg) for 24 hours, followed by dual luciferase reporter assays. **(M)** Assessing the Impact of NS2B^V35A^ on the IFN Reporter System: HEK293T cells were transfected with either empty vector or Flag-NS2B^V35A^, along with IFN-β luciferase reporter (IFN-β-Luc, 200 ng), PRL-TK (20 ng), and HA-RIG-I-(2CARD) (0.25 μg) for 24 hours, followed by dual luciferase reporter assays. **(N)** Assessing PP1α’s Impact on NS2B in the IFN Reporter System: HEK293T-PP1α^KO^ cells were transfected with either empty vector or Flag-NS2B, along with IFN-β luciferase reporter (IFN-β-Luc, 200 ng), PRL-TK (20 ng), and HA-RIG-I-(2CARD) (0.25 μg) for 24 hours, and subsequently subjected to dual luciferase reporter assays. **(O)** Vero cells were transfected with Flag-NS2B or infected with ZIKV for 24 hours, followed by As treatment. Immunostaining was performed, visualizing G3BP1 in green, anti-Flag or ZIKV-E in red. The symbol ’+’ denotes SG formation inhibition in NS2B-expressing and ZIKV-infected cells. A 10 μm white scale bar is included, and error bars represent standard deviation (n = 3). Statistical analysis used Student’s t-test, where ’n.s.’ signifies no significance, *P < 0.05, **P < 0.01, and ***P < 0.001.

While exploring the NS2B-PP1α-eIF2α axis’s role in suppressing SG formation, we aimed to decipher the critical role of PP1α within the ZIKV context. Surprisingly, when we expressed HA-PP1α, we observed its unexpected ability to inhibit ZIKV replication in both A549 and Hela cells (Fig 5A and 5B). Meanwhile, we observed an enhancement of ZIKV replication in HeLa-PP1α KO cells, which was efficiently inhibited upon exogenous replenishment of PP1α (Fig 5C and 5D). As previously reported [18,32], PP1α has the capacity to induce interferon (IFN) production. Our results confirmed that HA-PP1α can induce IFN production (Fig 5E), suggesting that PP1α has an antiviral effect on the replication of ZIKV. Altogether, in the context of IFN sufficiency, PP1α is ultimately reported to be antiviral.

To exclude the impact of IFN and elucidate PP1α’s proviral effect, we expressed HA-PP1α in Vero cells lacking of IFN signaling and observed HA-PP1α did enhance ZIKV replication in Vero cells (Fig 5F and 5G). Due to the absence of shRNA targeting monkey cells on the Addgene website, we designed sgRNA targeting Vero cells via the CHOPCHOP website, constructing a knockout cell line without monoclonal division, resulting in a mixed cell line (Vero-PP1α KO-mix cells) with functions equivalent to knockdown cells. In Vero-PP1α KO-mix cells, we observed a reduction in ZIKV replication compared to wild-type Vero cells (Fig 5H). Additionlly, the exogenous introduction of HA-PP1α into Vero-PP1α KO-mix cells led to an enhanced replication of ZIKV (Fig 5I). Furthermore, we assessed ZIKV replication in MEF-RIG-I^WT^ and MEF-RIG-I^KO^ cells, observing that PP1α inhibited ZIKV replication in MEF-RIG-I^WT^ cells but promoted its replication in MEF-RIG-I^KO^ cells (Fig 5J). These findings suggest that PP1α also acts as a pro-viral factor, highlighting that the dual nature of PP1α’s role in ZIKV infection. Considering the inhibitory effect of the NS2B-PP1α-eIF2α axis on SG formation and the reported capacity of NS2B to suppress IFN production [10,33] (Fig 5K and 5L), we examined whether there exists a relationship between NS2B’s impact on SGs and IFN production. Expressing NS2B^V35A^ revealed its ability to continue inhibiting IFN production (Fig 5M). Further observation demonstrated that NS2B also impeded IFN production in HEK293T-PP1α KO cells (Fig 5N), suggesting that NS2B’s inhibition of IFN production operates independently of its impact on SG formation. Similarly, we discovered that both NS2B and ZIKV infection can inhibit SG formation induced by As in Vero cells (Fig 5O), reinforcing that NS2B’s inhibition of SG formation is not dependent on its inhibition of IFN production. Collectively, our results emphasize the autonomous nature of the processes through which NS2B inhibits SG formation and IFN production.

### Broad-spectrum inhibition of SG formation by flavivirus NS2B

**Fig 6:**
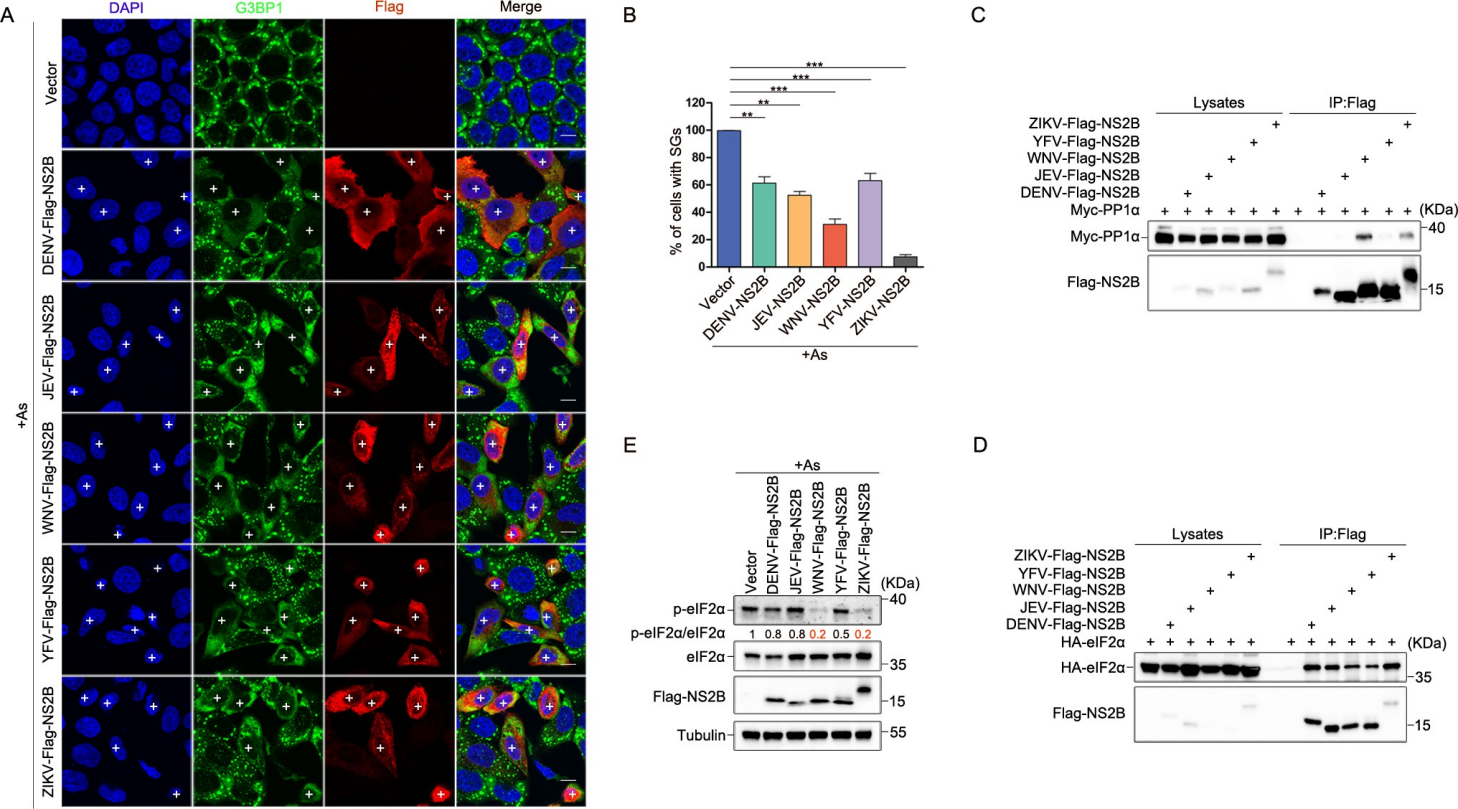
Flavivirus NS2B-mediated suppression of SG formation as a broad-spectrum antiviral approach. **(A and B)** Hela cells were transfected with empty vector, DENV-Flag-NS2B, JEV-Flag-NS2B, WNV-Flag-NS2B, YFV-Flag-NS2B and ZIKV-Flag-NS2B plasmids for 24 hours, followed by As treatment for 1 hour before harvesting. (A) Immunostaining was performed using anti-G3BP1 (in green) and anti-Flag (in red) antibodies; (B) The percentage of cells containing SGs was determined based on the analysis of three independent experiments. **(C and D)** HEK293T cells were transfected with Empty vector, DENV-Flag-NS2B, JEV-Flag-NS2B, WNV-Flag-NS2B, YFV-Flag-NS2B and ZIKV-Flag-NS2B plasmids, along with Myc-PP1α (C) or HA-eIF2α (D) for 36 hours. Subsequently, cell lysates were subjected to IP using anti-FLAG tag affinity gel. **(E)** Hela cells were transfected with empty vector, DENV-Flag-NS2B, JEV-Flag-NS2B, WNV-Flag-NS2B, YFV-Flag-NS2B and ZIKV-Flag-NS2B plasmids for 24 hours, followed by As treatment for 1 hour before harvesting. Cell lysates were subjected to western blot analysis using anti-phosphorylated eIF2α, anti-eIF2α, anti-Flag, and anti-tubulin antibodies. Cells that inhibited the formation of SGs were indicated by the ’+’ symbol. The white scale bar represents 10 μm, and error bars represent the standard deviation (n = 3). A total of 150 cells were counted in each experiment. Statistical analysis was carried out using Student’s t-test, where ’n.s.’ denotes not significant, *P < 0.05, **P < 0.01, and ***P < 0.001.

Building upon our earlier findings, we want to investigate whether NS2B proteins from other flaviviruses, notably DENV, JEV, WNV and YFV share the capacity to inhibit SG formation. The results confirmed that NS2B proteins from these flaviviruses indeed can inhibit SG formation (Fig 6A and 6B). Notably, NS2B from WNV was found to interact with both PP1α (Fig 6C) and eIF2α (Fig 6D), with the ability to dephosphorylate eIF2α (Fig 6E). Furthermore, we observed that NS2B from DENV, JEV, WNV, and YFV effectively inhibit SG formation in Hela-GADD34 KO cells (S5A and S5B Fig). The distinctive impact of WNV’s NS2B on SG formation was significantly diminished in Hela-PP1α KO cells (S5C and S5D Fig). Above all, these results together indicating that NS2B of WNV, similar to NS2B of ZIKV, can act as a bridge between PP1α and eIF2α to dephosphorylate eIF2α and inhibit SG formation. Conversely, NS2B proteins from DENV, JEV and YFV may employ distinctive mechanisms for inhibiting SG formation. Taken together, our results suggest that NS2B from flaviviruses exhibits a broad-spectrum inhibition of SG formation.

### NS2B^V35A^-mediated disruption of the PP1α-eIF2α axis alters ZIKV replication dynamics and cerebral organoid development

**Fig 7:**
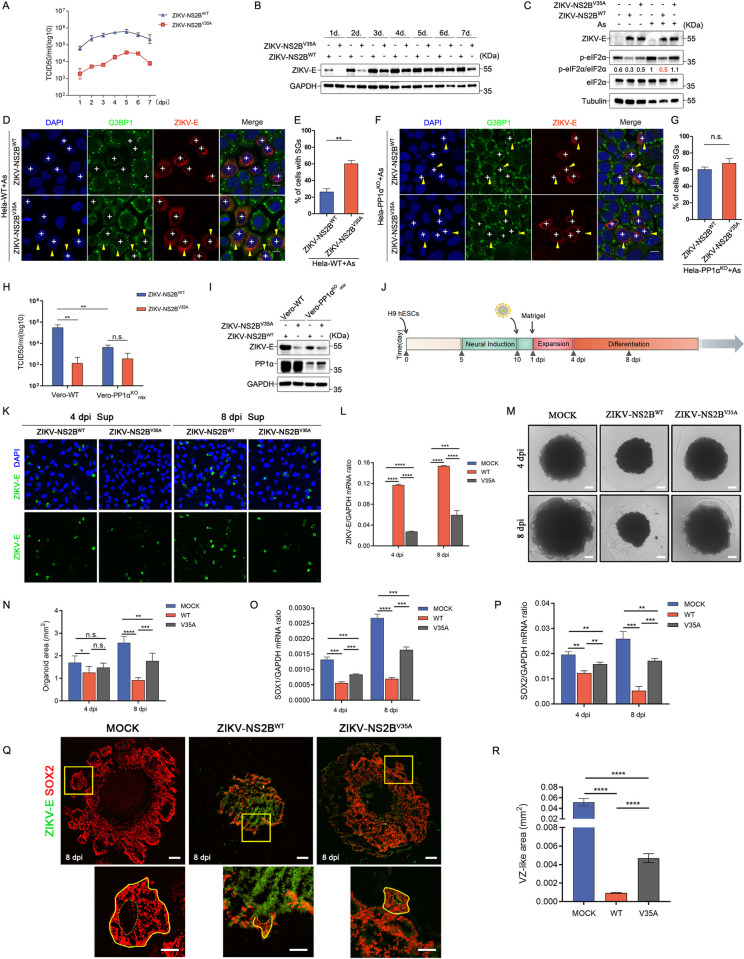
ZIKV-NS2B^V35A^ attenuates virus replication and mitigates neurotoxicity in brain organoid development. **(A and B)** Vero cells were infected with ZIKV-NS2B^WT^ and ZIKV-NS2B^V35A^ viruses at a MOI of 0.01. At 1, 2, 3, 4, 5, 6 and 7 dpi, (A) the supernatants were harvested for the TCID50 assay to establish virus growth curves, (B) and the cells lysates were analyzed for the ZIKV-E level through western blot analysis. **(C)** Hela cells were subjected to the following treatments: mock infection, ZIKV-NS2B^WT^ infection, and ZIKV-NS2B^V35A^ infection, with and without As treatment. Subsequently, cell lysates were analyzed through western blot. **(D and E)** Hela cells were infected with ZIKV-NS2B^WT^ and ZIKV-NS2B^V35A^, followed by As treatment. These cells were then subjected to immunostaining (D) and quantification of SGs (E). (Scale bars, 10μm). **(F and G)** Hela-PP1α^KO^ cells were infected with ZIKV-NS2B^WT^ and ZIKV-NS2B^V35A^, followed by As treatment. They were then subjected to immunostaining (F) and quantification of SGs (G). (scale bars, 10μm). **(H and I)** Vero-WT cells and Vero-PP1α^KO^mix cells were infected with ZIKV-NS2B^WT^ and ZIKV-NS2B^V35A^ viruses at a MOI of 0.01 for 2 days: (H) Supernatants were collected for the TCID50 assay, and (I) Cell lysates were analyzed for the ZIKV-E level via western blotting. **(J)** Diagram illustrating the viral exposure of 10-day-old brain organoids. **(K and L)** (K) Immunostaining of Vero cells incubated with supernatant (sup) of ZIKV-infected organoids at 4 and 8 dpi. DAPI (blue) was used to stain nuclear DNA. Scale bars, 75μm. (L) qRT-PCR analysis of ZIKV envolope (ZIKV-E) RNA in organoids exposed to ZIKV. **(M and N)** Images (M) (scale bars, 500μm) and area measurements of organoids (N) exposed to ZIKV-NS2B^WT^, ZIKV-NS2B^V35A^ or mock-treated. **(O and P)** qRT-PCR analysis of SOX1 (O) and SOX2 (P) in organoids exposed to ZIKV-NS2B^WT^, ZIKV-NS2B^V35A^ or mock-treated. **(Q)** Immunostaining of organoids exposed to ZIKV-NS2B^WT^, ZIKV-NS2B^V35A^ or mock-treated (scale bars, 100μm). Insets in (Q) (scale bars, 50μm) exhibit the ventricular zone (VZ)-like structures (full lines) and their lumens (dashed lines). **(R)** The measurement of VZ-like structures of organoids exposed to ZIKV-NS2B^WT^, ZIKV-NS2B^V35A^ or mock-treated. Cells with the symbol ’+’ represent ZIKV-infected cells that exhibited inhibition of SG formation, while cells with both ’+’ and yellow arrowheads indicate ZIKV-infected cells that failed to inhibit SG formation. Error bars represent the standard deviation of data obtained from three independent experiments, with 150 cells counted each time. Statistical analysis was performed using Student’s t-test, where n.s. denotes no statistical significance, *P < 0.05 denotes statistical significance at the 5% level, **P < 0.01 denotes statistical significance at the 1% level, and ***P < 0.001 denotes statistical significance at the 0.1% level.

To further elucidate the role of NS2B inhibiting SG formation on ZIKV production, we embarked on the construction of a functionally impaired recombinant virus, ZIKV-NS2B^V35A^. Our investigation scrutinized the replication dynamics of wild-type ZIKV (ZIKV-NS2B^WT^) and ZIKV-NS2B^V35A^ in Vero cells, illuminating a substantial reduction in the replication of ZIKV-NS2B^V35A^ compared to ZIKV-NS2B^WT^ (Fig 7A and 7B). Subsequently, we probed the dephosphorylation capabilities of ZIKV-NS2B^V35A^ and found its failure to dephosphorylate eIF2α (Figs 7C and S6A). Correspondingly, the ability of ZIKV-NS2B^V35A^ to suppress SG formation exhibited significant attenuation (Figs 7D, 7E, S6B and S6C). Moreover, in Hela-PP1α KO cells and Vero-PP1α KO-mix cells, we detected no significant difference in the ability of ZIKV-NS2B^V35A^ and ZIKV-NS2B^WT^ to inhibit SG formation (Figs 7F, 7G, S6D and S6E). Furthermore, the replication of ZIKV-NS2B^WT^ was significantly hampered in Vero-PP1α KO-mix cells compared to wild-type cells. In contrast, ZIKV-NS2B^V35A^ and ZIKV-NS2B^WT^ exhibited no notable difference in replication within Vero-PP1α KO-mix cells (Fig 7H and 7I). These results collectively suggest that ZIKV-NS2B^V35A^, through its disruption of the NS2B-PP1α-eIF2α axis, mitigates eIF2α dephosphorylation and its inhibitory effect on SG formation, consequently diminishing ZIKV-NS2B^V35A^ replication.

Given the extensive application of human cerebral organoids in modeling ZIKV infection-associated microcephaly, we further delved into the comparative impacts of ZIKV-NS2B^WT^ and ZIKV-NS2B^V35A^ infection during the early stages of human brain development. Our research focused on day 10 cerebral organoids, which were subjected to viral infection for 24 hours (Fig 7J). After inoculum removal, organoids were examined on day 4 and day 8 post-infection. The increasing expression of ZIKV-E RNA and the release of infectious particles were detected on day 4 and day 8 following infection with both ZIKV-NS2B^WT^ and ZIKV-NS2B^V35A^ (Fig 7K and 7L), which indicated valid and productive infection. Notably, compared with mock-treated organoids, brain organoids infected by ZIKV-NS2B^WT^ exhibited a time-dependent reduction in growth (Fig 7M and 7N). In striking contrast, organoids infected with ZIKV-NS2B^V35A^ were notably larger than their ZIKV-NS2B^WT^-infected counterparts on day 8 (Fig 7M and 7N). Considering that human neural progenitor cells (hNPCs) are the primary targets of ZIKV infection, and cerebral organoids encompass areas resembling the ventricular zone (VZ) where hNPCs predominantly reside, we firstly evaluated the RNA levels of hNPC markers SOX1 and SOX2 on day 4 and day 8 post-infection. The findings indicated that the RNA levels of SOX1 and SOX2 in brain organoids infected by both viruses were significantly lower than those in mock-treated organoids (Fig 7O and 7P). However, the SOX1 and SOX2 RNA levels in ZIKV-NS2B^V35A^-infected organoids were significantly higher than those in ZIKV-NS2B^WT^-infected organoids (Fig 7O and 7P). Furthermore, we conducted staining for SOX2^+^ hNPCs in the VZ-like region to directly observe virus-induced depletion of hNPCs. This analysis revealed that ZIKV-NS2B^WT^-infected organoids displayed fewer and smaller VZ-like structures than mock-treated organoids (Fig 7Q and 7R). While ZIKV-NS2B^V35A^-infected organoids were also smaller than mock-treated organoids, they exhibited more substantial and complete morphological features of VZ-like structures compared to ZIKV-NS2B^WT^-infected organoids (Fig 7Q and 7R). Additionally, we assessed apoptosis levels between ZIKV-NS2B^WT^ and ZIKV-NS2B^V35A^-infected organoids, finding that ZIKV-NS2B^V35A^ displayed lower levels of apoptosis, as evidenced by staining for the apoptotic marker cleaved caspase-3 (CC3) (S7A Fig). In summation, these results underscore that ZIKV-NS2B^V35A^ mitigates its toxicity to organoid growth, abates hNPC depletion, and reduces the manifestation of a microcephaly-like phenotype during the early stages of human brain organoid development in comparison to ZIKV-NS2B^WT^ infection.

## Discussion

Flaviviruses have been found to influence SG formation through two primary mechanisms: (1) by hijacking essential components of SGs. For instance, the C protein of JEV impedes SG formation by sequestering Caprin1, G3BP1 and TIA-1 [34]. Additionally, the 3’-stem loop structure within the minus chain of the DENV and WNV genomes can hinder SG formation by hijacking TIA-1 and TIAR [35]. Similarly, the ZIKV disrupts SG formation by commandeering Caprin1 and G3BP1 [23,24]. (2) Through the inhibition of eIF2α phosphorylation. Notably, ZIKV increases the expression of GADD34, which, in turn, dampens eIF2α phosphorylation [25]. Furthermore, JEV NS2A and HCV NS5A impede eIF2α phosphorylation by preventing the dimerization of PKR [36,37]. WNV, DENV, Usutu Virus (USUV) and others have also been documented to inhibit eIF2α phosphorylation [26,35,38,39]. However, the precise mechanism through which flaviviruses dephosphorylate eIF2α remains a topic of ongoing investigation.

In this study, we investigated the precise mechanism by which the ZIKV modulates eIF2α dephosphorylation, leading to the inhibition of SG formation. Initially, we observed that ZIKV did not induce SG formation in Hela and A549 cells (Figs 1A, 1B, S1A, S1B, S1E and S1F). Subsequently, we demonstrated that ZIKV exerted inhibitory effects on SG formation (Figs 1C–1F, S1C, S1D, S1G and S1H) and eIF2α phosphorylation (Fig 1G–1J). Moreover, we discovered that ZIKV NS2B played a critical role in inhibiting both SG formation (Fig 1K–1N) and eIF2α phosphorylation (Fig 1O–1Q). Two possible mechanisms could explain how ZIKV NS2B inhibits eIF2α phosphorylation, namely eIF2α cannot be phosphorylated or eIF2α is dephosphorylated. Our result detected the activation of PKR during ZIKV infection (S2A Fig), and since PKR is known to directly phosphorylate eIF2α [40,41], it suggests that NS2B dephosphorylates eIF2α. To further investigate the mechanism through which NS2B dephosphorylates eIF2α, we performed IP/MS analysis and observed a specific interaction and co-localization of NS2B with PP1α (Figs 2A, 2D, 3D and S3A). This interaction was critical in the dephosphorylation process. The significance of PP1α in NS2B-mediated eIF2α dephosphorylation was further validated through KO of PP1α and subsequent complementation of PP1α (Figs 2E, 2F, S3B and S3C). Additionally, functional deficiency analyses of NS2B^V35A^ (S3D–S3H Fig) further supported that the essential role of PP1α in NS2B-mediated eIF2α dephosphorylation and SG formation inhibition. Consequently, the ability of NS2B to inhibit SG formation was significantly impaired in PP1α KO cells compared to WT cells (Fig 2G and 2H). These findings highlight the critical role of the NS2B-PP1α axis in modulating eIF2α phosphorylation and SG formation during ZIKV infection.

GADD34/PP1 plays a critical role in the innate immune response by facilitating the dephosphorylation of key proteins involved in regulating IFN production. These proteins encompass MDA5/RIG-I, TAK1, TBK1, and IKKβ [18,32]. Our research further explores the role of NS2B as an intermediary connecting PP1α and eIF2α, thus facilitating the dephosphorylation of eIF2α in a GADD34-independent manner (Figs 3 and S4). However, we cannot rule out the potential involvement of other cytokines in this process. Furthermore, we observed that NS2B and PP1α engaged in a reciprocal inhibition of K48-linked ubiquitination, effectively stabilizing their expression (Fig 4). Meanwhile, our study revealed the dual function of PP1α in ZIKV infection. On one hand, PP1α exerted an inhibitory effect on ZIKV replication by stimulating IFN production (Fig 5A–5D). On the other hand, PP1α can also promote ZIKV replication by dephosphorylating eIF2α and hampering the formation of SGs (Fig 5F–5J). This duality underscores the multifaceted role of PP1α during ZIKV infection, where it acts as a double-edged sword, influencing both the antiviral immune response and viral replication dynamics.

Earlier investigations have established that NS2B is capable of obstructing the production of IFN-β [10,33]. In some viral systems, such as the Nipah virus, the binding of viral proteins to PP1 can thwart IFN production by competing with MDA5 for PP1 binding [42,43]. This led us to explore whether NS2B’s inhibition of IFN production is mediated through its interaction with PP1α. However, our results clearly indicated that the inhibition of IFN production by NS2B was independent of PP1α (Fig 5M and 5N). In conclusion, we have provided evidence demonstrating that NS2B was capable of independently impeding the formation of SGs and IFN production (Fig 5K–5O). Additionally, our investigations proved that the inhibitory effect of flavivirus NS2B on SG formation was a broadly applicable phenomenon, with a similar mechanism of NS2B’s ability to hinder SG formation in WNV and ZIKV (Figs 6 and S5). WNV NS2B shares a high degree of structural similarity with ZIKV NS2B [44]. These findings shed light on a shared strategy among flaviviruses in modulating the cellular stress response.

To further investigate the intricate regulatory axis governed by NS2B in ZIKV infection, we sought to elucidate the specific role of NS2B^V35A^, a non-interacting variant that failed to engage with PP1α, dephosphorylate eIF2α, and inhibit SG formation. Our primary objective was to establish how NS2B, in conjunction with PP1α and eIF2α, regulates SG formation, subsequently influencing virus replication dynamics under ZIKV infection conditions. To this end, we ingeniously introduced NS2B^V35A^ into the viral genome to replace NS2B, consequently generating a recombinant mutant virus termed ZIKV-NS2B^V35A^. The functional limitations of this mutant virus were abundantly clear, as its capacity to dephosphorylate eIF2α (Figs 7C and S6A) and inhibit SG formation (Figs 7D, 7E, S6B and S6C) were substantially compromised, attributed to the inability of NS2B^V35A^ to interact with PP1α. Therefore, this translated into a weakened replication ability of ZIKV-NS2B^V35A^ due to its ineffectiveness in inhibiting SG formation. Strikingly, we also provided evidence that when PP1α was knocked out in cells, there was no significant difference in the replication ability between ZIKV-NS2B^V35A^ and the wild-type ZIKV (Fig 7H and 7I). This underscored the pivotal role of the NS2B-PP1α-eIF2α axis in orchestrating the regulation of ZIKV replication. These findings collectively underscore the network of interactions governing ZIKV replication, where the NS2B-PP1α-eIF2α axis plays a central role in dephosphorylating eIF2α, modulating SG formation and, consequently, viral propagation.

In previous investigations, brain organoid models, sophisticated three-dimensional cultures that mimic organogenesis, have proven invaluable for exploring the pathogenic mechanisms of Zika Virus (ZIKV) infection [45–47]. It is well-established that ZIKV infection can lead to heightened neural cell apoptosis and the development of microcephaly, a condition characterized by an abnormally small head and incomplete brain development [48,49]. To emulate the physiological role of NS2B within the context of ZIKV infection, we harnessed virus-infected organoids as a model to delve into the pathogenicity of ZIKV infection. Our results revealed that ZIKV-NS2B^WT^ had a discernible impact on organoid growth, resulting in a depletion of hNPCs, elevated levels of apoptosis, and a more pronounced microcephaly-like phenotype in the early stages of human brain organoids compared to ZIKV-NS2B^V35A^ infection (Figs 7J–7R and S7A). These findings underscore the important role of the NS2B-PP1α-eIF2α axis in dictating the pathogenicity of ZIKV infection, further emphasizing the significance of this regulatory axis in the context of neural development and disease progression.

**Fig 8:**
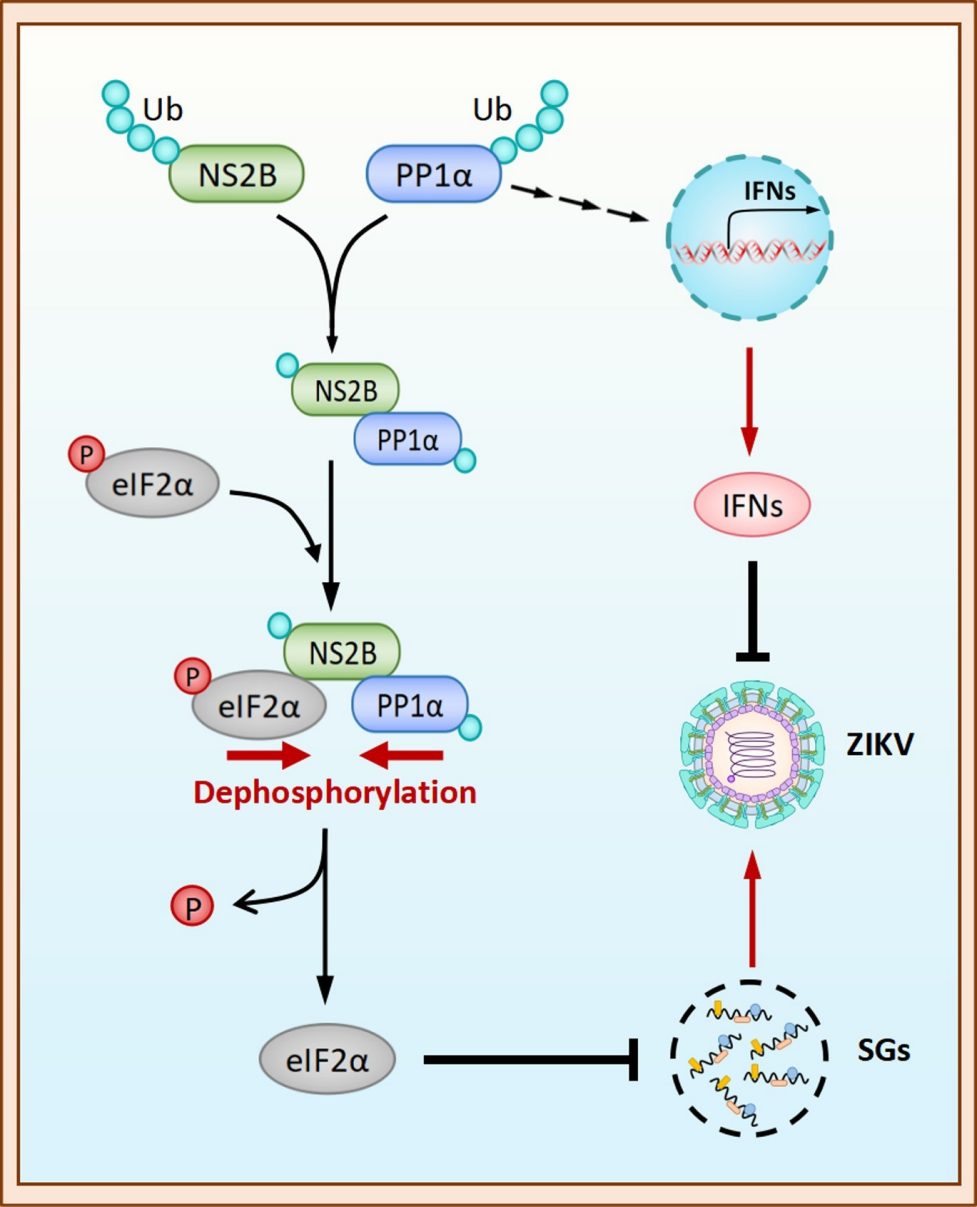
A schematic illustration of the NS2B-PP1α-eIF2α axis inhibiting SG formation during ZIKV infection. In Zika virus-infected cells, NS2B acts as a bridge, facilitating the interaction between PP1α and eIF2α. This interaction leads to eIF2α dephosphorylation, inhibiting SG formation and promoting viral replication. Simultaneously, PP1α induces interferon production, serving both antiviral and proviral functions during the viral replication process.

In summary, our investigation reveals a new and distinctive function of NS2B, enriching the understanding of intricate interactions between viral components and host factors in flavivirus infections. Our study elucidates the mechanism through which NS2B utilizes PP1α to dephosphorylate eIF2α, hence promoting ZIKV replication (Fig 8). These findings emphasize the versatile role of PP1α in the landscape of viral pathogenesis (Fig 8). The conspicuous absence of effective vaccines against flaviviruses underscores the pressing need to develop precisely targeted antiviral therapies. Our research, therefore, offers a rationale for the development of antiviral drugs designed to selectively target the NS2B-PP1α-eIF2α axis. Such therapeutic strategies hold great promise for the treatment of diseases induced by flaviviruses, potentially opening up new avenues to combat these medically significant pathogens.

## Materials and methods

### Cell culture

The cell lines employed in this study encompass HEK293T cells, HEK293T cells with a KO of PP1α, Hela cells, Hela cells with KO of PP1α, Hela cells with KO of GADD34, A549 cells, Vero cells, and Vero cells with a knockout of PP1α-mix (referred to as Vero-PP1α KO mix cells). MEF-RIG-I^WT^ and MEF-RIG-I^KO^ cells were obtained from the laboratory of Professor Hongbing Shu (Wuhan University). These cell lines were meticulously maintained in Dulbecco’s modified Eagle’s medium (DMEM), augmented with 10% fetal bovine serum (FBS) and 1% penicillin-streptomycin. Cultivation was carried out at a temperature of 37°C within a humidified incubator with 5% CO_2_, ensuring optimal conditions for cell growth and experimental reproducibility.

### Reagents and antibodies

In this study, several antibodies were used for immunoblotting and immunoprecipitation experiments. GADD34 polyclonal antibody (catalogue no. A16260), rabbit polyclonal anti-PABP antibody (catalogue no. A14872), rabbit polyclonal anti-EIF4G1 (catalogue no. A7552), rabbit polyclonal anti-G3BP1 (catalogue no. A3968) and rabbit anti-eIF4E antibody (catalogue no. A2162) were purchased from Abclonal. Additionally, PPP1CA Rabbit polyclonal antibodies were purchased from abclonal (catalogue no. A12468) and proteintech (catalogue no. 67070-1-Ig). PKR antibody (catalogue no. 3072S), Phospho-eIF2α (Ser51) Antibody (catalogue no. 9721S), DYKDDDDK Tag (D6W5B) Rabbit monoclonal antibody (catalogue no. 14793S), HA-Tag (C29F4) Rabbit monoclonal antibody (catalogue no. 3724S), RIG-I (D14G6) Rabbit monoclonal antibody (catalogue no. 3743T) and Myc-Tag (71D10) Rabbit monoclonal antibody (catalogue no. 2278S) were purchased from CST. The eIF2α Rabbit polyclonal antibody was purchased from abclonal (catalogue no. A0764) and CST (catalogue no. 9722S). Rabbit anti-phosphorylated PKR antibody (catalogue no. ab81303) was purchased from Abcam, and goat anti-TIA-1 was purchased from ThermoFisher (catalogue no. PA5-18699). Zika virus Envelope protein (GTX634155) was purchased from GeneTex. Poly(I:C) was purchased from InvivoGen.

### KO cell lines construction

HEK293T-PP1α KO cells were meticulously generated through a well-defined procedure. Initially, these cells were transiently transfected with the PX459-KO-PP1α plasmid, a process extending over 48 hours. Following transfection, the cells underwent selection, which involved exposure to 1 μg/ml of puromycin for a duration of 72 hours. Surviving cells were subsequently subjected to a sorting process to isolate and establish a monoclonal cell line. The guide RNA (sgRNA) employed to target human PP1α was designed with the sequence GAGAACTTCTTCCTGCTCCG. The generation of Vero-PP1α KO-mix cells involved a slightly distinct methodology. Initially, lentivirus was produced by transfecting HEK293T cells with lentiCRISPRv2 containing PP1α-targeting sgRNA (with the sequence GGCGCCCGATGATCGAGTCA), psPAX2, and pMD2.G. This transfection process spanned 48 hours. Subsequently, the lentivirus supernatant was harvested, and cellular debris was effectively removed through filtration using a 0.45-μm Millipore filter. Vero cells were then subjected to infection with the lentivirus expressing the sgRNA, followed by selection with 6 mg/ml of puromycin for a period of one week to yield the Vero-PP1α KO-mix cells. It’s important to note that Hela cells with KO of PP1α and Hela cells with KO of GADD34 were procured directly from the EDITGENE company, representing a valuable resource for the study. These carefully established cell lines and their respective knockout procedures played an integral role in the experimental design and data interpretation.

### Viral infection and titration

In preparation for the viral infection experiments, HeLa cells were thoughtfully seeded into 6-well, 12-well, or 24-well plates at a density approximating 40–50%. Meanwhile, A549 and Vero cells were cultured at a higher density, typically ranging between 80–90%. Following this initial setup, the cells were subjected to infection with the ZIKV for a designated duration of 2 hours. Subsequent to the infection phase, the culture medium was judiciously replaced with fresh DMEM, thoughtfully fortified with 8% fetal FBS.

In order to ascertain the 50% tissue culture infectious dose (TCID50), a systematic procedure was implemented. Vero cells were initially seeded into 96-well plates at a controlled density, typically falling within the range of 20–30%. Following this setup, the subsequent steps were undertaken with precision. The assessment involved the careful observation of cytopathic effects (CPE) in each well at various dilutions. The count of wells exhibiting these CPE served as the basis for quantifying the viral titers. The calculations were performed in adherence to the Karber method.

### SGs inducion and drug treatment

HeLa cells were subject to treatment with a concentration of 200 μM of As, a compound sourced from Sigma-Aldrich, for a duration of 1 hour. Conversely, A549 or Vero cells were exposed to a slightly higher concentration of 500 μM of As, also for a duration of 1 hour. Hela cells were subjected to a 1-hour treatment with 2 nM of DTT, or a 12-hour treatment with Poly(I:C). Subsequently, the quantification of SGs was conducted by systematically counting at least 150 cells within each group.

### Western blot and immunoprecipitation analysis

In the course of Western blot analysis, the following procedure was followed: Cells were collected and lysed in lysis buffer, which contained 150 mM NaCl, 50 mM Tris-HCl (pH 7.4), 1% Triton X-100, 1 mM EDTA (pH 8.0), and 0.1% sodium dodecyl sulfate (SDS). The lysis buffer was additionally fortified with a protease inhibitor cocktail to ensure the preservation of protein integrity. The lysate was subsequently subjected to an incubation period on ice, lasting 30 minutes. Following this incubation, the lysate was centrifuged at 12,000 g at 4°C for a duration of 30 minutes, facilitating the collection of the supernatant. The protein concentration was quantified using the Bradford assay method. Subsequently, the protein samples were thoroughly mixed with SDS-PAGE loading buffer and then subjected to boiling at 100°C for a period of 10 minutes. The protein samples were then loaded onto SDS polyacrylamide gels with varying percentages (ranging between 8%, 10%, or 11%) to facilitate electrophoresis. The proteins were subsequently transferred from the gel onto nitrocellulose membranes. To prevent nonspecific binding, the nitrocellulose membrane was effectively blocked using a solution comprising 5% milk in PBS with 0.1% Tween 20. This blocking step lasted for a duration of 30 minutes. Following membrane blocking, the nitrocellulose membrane was then incubated with primary antibodies overnight, which allowed for the specific recognition of target proteins within the sample. Subsequently, the membrane was subjected to incubation with secondary antibodies for a period of 1 hour. The secondary antibodies used included HRP-conjugated goat anti-mouse IgG and goat anti-rabbit IgG, both at a dilution of 1:5000.

For immunoprecipitation, cells overexpressing the targeted proteins were subjected to lysis using a specialized lysis buffer. Subsequently, the supernatants were carefully collected. To minimize non-specific interactions, the collected supernatants were prepped by undergoing a pre-clearing step. This involved their incubation with protein G Sepharose 4 Fast Flow beads for a duration of 2 hours at 4°C, with regular rotation. Following this incubation, centrifugation was carried out to separate the beads from the supernatants. Specific primary antibodies, designed to selectively target the proteins of interest, were thoughtfully introduced into the supernatants. The mixture was then subjected to an overnight incubation at 4°C, with consistent rotation. Following the antibody incubation, the protein-antibody complexes were precipitated by collecting the beads. Subsequent to bead collection, a thorough washing procedure was performed. This involved three sequential washes with a specialized buffer, composed of 5% (wt/vol) sucrose, 5 mM Tris-HCl (pH 7.4), 5 mM EDTA (pH 8.0), 500 mM NaCl, and 1% (vol/vol) Triton X-100. Finally, the collected beads were subjected to boiling at 100°C for 10 minutes in 2× SDS protein loading buffer. The resulting protein samples were then analyzed by Western blotting, allowing for the specific identification and quantification of protein-protein interactions.

### Immunofluorescence assay

To perform immunofluorescence, HeLa, A549, or Vero cells were cultured on coverslips in 24-well plates. They were either transfected with appropriate plasmids using Lipofectamine 2000 reagent (Invitrogen) or infected with ZIKV. After specific time intervals, the cells were fixed with 4% paraformaldehyde in 1×PBS for 20 minutes at room temperature and washed three times with PBS for 5 minutes each. The cells were then permeabilized with 0.2% Triton X-100 for 20 minutes and blocked with 3% BSA. Organoids were fixed with 4% paraformaldehyde (PFA) in 1×PBS at 4°C overnight, immersed in a 30% sucrose solution overnight at 4°C, embedded in O.C.T. compounds, and frozen. Organoid blocks were sectioned at 10 μm thickness using a freezing microtome (Leica). Organoid cryosections were fixed with 4% PFA for 15 minutes and washed with 1×PBS extensively. For immunofluorescence staining, primary antibodies, appropriately diluted in 1% BSA, were incubated overnight at 4°C. Secondary antibodies, also diluted in 1% BSA, were added for 1 hour at room temperature. Nuclei were counterstained with DAPI for 10 minutes, and cells were mounted with Prolong Diamond Antifade Mountant (Life Technology) before examination using a Leica confocal microscope.

### Luciferase reporter gene assays

HEK293T cells were seeded in 24-well plates at 50%−60% confluence and transfected with a mix of 20 ng pRL-TK (Renilla luciferase plasmid) and 200 ng of a luciferase reporter plasmid (firefly luciferase), along with either a specified expression plasmid or an empty vector. Following this, HEK293T cells were co-transfected with RIG-I(2CARD) for 24 hours or stimulated with poly(I:C) for 12 hours. Luciferase activity was measured using the Dual-Luciferase Reporter Assay System kit from Promega (San Luis Obispo, CA) following the manufacturer’s instructions. Experiments were performed in triplicate and repeated at least three times. The data represents firefly luciferase activity normalized to Renilla luciferase activity.

### Generation of cerebral organoids

Human cerebral organoids were derived from human-induced pluripotent stem cells (hiPSCs), following a previously established protocol. In brief, on day 0, hiPSCs were enzymatically dissociated into individual cells using accutase. Approximately 2000 cells were then seeded into hanging drop culture plates (InSphero AG) to form single embryoid bodies (EBs) over the course of one day. Subsequently, these EBs were transferred to ultra-low-attachment 96-well plates (Corning) and cultured in an EB formation medium with a low concentration of basic fibroblast growth factor (bFGF) at 4 ng/ml, along with a ROCK inhibitor at 50 mM. On day 3, the culture medium was refreshed to fresh stem cell medium. By day 6, the medium was replaced daily with Neural Induction Medium, comprising DMEM/F12 (Life Technologies), 1× N2 supplement (Life Technologies), 1% non-essential amino acids (Life Technologies), 2 mM GlutaMAX, and 1 μg/ml heparin. On day 11, the EBs were gently embedded into Matrigel droplets and subsequently transferred to 6-cm dishes containing neural expansion medium. This medium was composed of 50% DMEM/F12, 50% Neurobasal medium, 0.5× N2 supplement, 0.5× B27 supplement (without vitamin A), 2 mM GlutaMAX, 2.5 ng/ml human insulin, 0.5% non-essential amino acids (Life Technologies), and 25 nM beta-mercaptoethanol (Life Technologies). Upon reaching day 15, the culture medium was transitioned to a neural maturation medium, which consisted of 50% DMEM/F12, 50% neurobasal medium, 0.5× N2 supplement, 0.5× B27 supplement (with vitamin A, Life Technologies), 2 mM GlutaMAX, 2.5 ng/ml human insulin, 0.5% non-essential amino acids, and 25 nM beta-mercaptoethanol. Organoids were cultured on an orbital shaker, rotating at 75 rpm, and the medium was refreshed every two days.

### Infection of cerebral organoids

On the tenth day of cultivation, the cerebral organoids were subjected to an incubation with either ZIKV-NS2B^WT^ or ZIKV-NS2B^V35A^. This incubation was carried out with the viral components diluted in 200 μl of neural induction medium. The organoids were placed in ultra-low binding 96-well plates, as depicted in the Fig 7J These plates were then incubated within a 5% CO_2_ environment at 37°C. Following a one-day infection period, the organoids were embedded in Matrigel and subsequently transferred to 6-cm dishes containing the expansion medium. They were maintained and cultured in accordance with the previously described procedures.

### Generation of recombinant ZIKV-NS2B^V35A^

The pFLZIKV-FSS13025 mutant plasmid underwent amplification within E. coli Top10 and was subsequently purified employing MaxiPrep PLUS (QIAGEN). For the purpose of in vitro transcription, a 10 μg plasmid was linearized using the ClaI restriction enzyme, and in vitro transcription of RNA was accomplished using the mMESSAGE T7 kit (AM1344, Ambion) in strict accordance with the kit’s provided instructions. Following transcription, the RNA was precipitated using lithium chloride and quantified through spectrophotometry. To facilitate transfection, 10 mg of RNA was electroporated into Vero cells within 4 mm cuvettes, employing the GenePulser apparatus from Bio-Rad. Subsequently, after a 10-minute recuperation period at room temperature, the transfected cells were mixed with DMEM in T75 flasks and placed within a 5% CO_2_ incubator at 37°C. Recombinant viruses present in the cell culture media were harvested after a 4-day period following transfection.

### Statistical analysis

Statistical analysis to evaluate the significance of variability among different groups was conducted through two-way analysis of variance (ANOVA) tests utilizing GraphPad Prism software, version 5.0. The entire statistical analysis was executed with GraphPad Prism, specifically version 8.02. The data is expressed as means ± standard deviation (SD) and originates from a minimum of three independent experiments. The calculation of p-values was carried out employing an unpaired Student’s t-test, with a threshold of p<0.05 considered as statistically significant, as denoted by the following notation: *, p<0.05; **, p<0.01; ***, p<0.001. In instances where p>0.05, it was determined to be statistically non-significant and indicated as (n.s.).

## Supporting information

S1 Fig Cell-specific analysis of viral infection effects on SG formation using multiple marker proteins. **(A)** Immunofluorescence analysis was performed to visualize protein aggregation in Hela cells that were either mock-infected or infected with ZIKV (MOI of 0.3) for 36 hours. **(B)** The percentage of cells containing SGs was quantified in three independent experiments based on the observations in panel (A). **(C)** Hela cells were mock-infected or infected with ZIKV (MOI of 0.3) for 36 hours, followed by treatment with As (200uM,1h). **(D)** The percentage of cells containing stress granules was quantified in three independent experiments based on the observations in panel (**C**). **(E)** A549 cells were mock-infected or infected with ZIKV (MOI of 0.1) for 24 hours, and then subjected to immunofluorescence staining to visualize G3BP1 (green) and ZIKV-E (red). **(F)** The percentage of cells containing stress granules was quantified in three independent experiments based on the observations in panel (E). **(G)** A549 cells were mock-infected or infected with ZIKV (MOI of 0.1) for 24 hours, followed by treatment with As (500uM,1h). **(H)** The percentage of cells containing stress granules was quantified in three independent experiments based on the observations in panel (**G**). Cells labeled with "+" indicate ZIKV-infected cells that inhibited SG formation, while cells marked with both "+" and yellow arrowheads indicate those that did not. The white scale bar represents a length of 10 μm. Data is presented as mean ± SD (n = 3), with a total of 150 cells counted in each experiment. Statistical analysis was conducted using Student’s t-test, where n.s. indicates no significant difference, and *P < 0.05, **P < 0.01, ***P < 0.001 indicate significant differences.
(TIF)

S2 Fig PKR activation during ZIKV Infection. **(A)** Western blot analysis depicting the activation state of PKR during ZIKV infection.
(TIF)

S3 Fig NS2B-mediated dephosphorylation requires PP1α involvement. **(A)** Fluorescence intensity profiles of Myc-PP1α, Myc-PP1β, Myc-PP1γ, and Flag-NS2B in Hela cells transfected with Myc-PP1α, Myc-PP1β, Myc-PP1γ, and Flag-NS2B for 24 hours. **(B)** Western blot analysis comparing PP1α protein expression levels between HEK293T WT cells and HEK293T-PP1α^KO^ cell clones. **(C)** Western blot analysis of HEK293T-PP1α^KO^ cells transfected with empty vector, Flag-NS2B, or Flag-NS2B and HA-PP1α with synonymous mutations, followed by treatment with or without As. **(D)** Schematic diagrams of N-terminally truncated or point mutants of NS2B. **(E and F)** Immunostaining (E) and quantification of SGs (F) of N-terminally truncated or point mutants of NS2B. **(G)** Western blot analysis of HEK293T cells transfected with empty vector, Flag-NS2B, or Flag-NS2B^V35A^, followed by treatment with or without As. **(H)** Immunoprecipitation assays comparing the interaction of NS2B, NS2B^V35A^, and HA-PP1α in HEK293T cells. The symbol "+" denotes cells expressing NS2B or its mutants that inhibited SG formation, while cells with both "+" and yellow arrowheads indicate cells expressing NS2B or its mutants that failed to inhibit SG formation. The white scale bar represents a length of 10 μm. The data is presented as mean ± SD (n = 3). In each experiment, 150 cells were counted. Statistical analysis was conducted using Student’s t-test, with n.s. indicating non-significant results, and *P < 0.05, **P < 0.01, ***P < 0.001 denoting significant differences.
(TIF)

S4 Fig NS2B colocalizes with PP1α and eIF2α independently of GADD34. **(A and C)** Hela cells (A) and Hela-GADD34^KO^ cells (C) were transfected with Myc-PP1α, Flag-NS2B, Myc-PP1α and Flag-NS2B, Flag-NS2B^V35A^, Myc-PP1α and Flag-NS2B^V35A^ plasmids for 24 hours. The cells were subsequently immunostained with anti-Myc (green) and anti-Flag (red) antibodies, and the fluorescence intensity profile of PP1α (green) and NS2B, NS2B^V35A^ (red) was measured. A white scale bar of 10 μm was included for reference. **(B and D)** Hela cells (B) and Hela-GADD34^KO^ cells (D) were transfected with Myc-eIF2α, Flag-NS2B, Myc-eIF2α and Flag-NS2B, Flag-NS2B^V35A^, Myc-eIF2α and Flag-NS2B^V35A^ plasmids for 24 hours. The cells were immunostained with anti-Myc (green) and anti-Flag (red) antibodies, and the fluorescence intensity profile of eIF2α (green) and NS2B, NS2B^V35A^ (red) was measured. A white scale bar of 10 μm was included for reference.
(TIF)

S5 Fig Diverse mechanisms employed by flavivirus NS2B to suppress SG Formation. **(A and B)** Investigation of Flavivirus NS2B’s influence on SG formation in Hela-GADD34^KO^ cells. Cells were transfected with either empty vector or plasmids encoding DENV-Flag-NS2B, JEV-Flag-NS2B, WNV-Flag-NS2B, YFV-Flag-NS2B and ZIKV-Flag-NS2B for 24 hours, followed by a 1-hour treatment with As before harvesting. (A) Immunostaining with anti-G3BP1 (green) and anti-Flag (red) antibodies; (B) Analysis of the percentage of cells containing SGs from the experiments in panel (A). **(C and D)** Hela-PP1α^KO^ cells were transfected with empty vector, DENV-Flag-NS2B, JEV-Flag-NS2B, WNV-Flag-NS2B, YFV-Flag-NS2B and ZIKV-Flag-NS2B plasmids for 24 hours and subsequently treated with As for 1 hour before harvesting; (C) Immunostaining with anti-G3BP1 (green) and anti-Flag (red); (D) Analysis of the percentage of cells containing SGs from the experiments in panel (C) Cells with the symbol “+” represent cells expressing NS2B that effectively inhibit SG formation, while cells marked with both “+” and yellow arrowheads indicate NS2B-expressing cells that fail to inhibit SG formation. The white scale bar indicates 10 μm. Error bars denote the standard deviation of results from three independent experiments, with 150 cells counted in each experiment. Statistical significance was determined using Student’s t-test, where n.s. indicates no significance, *P < 0.05, **P < 0.01, and ***P < 0.001.
(TIF)

S6 Fig Disruption of NS2B-PP1α interaction weakens eIF2α dephosphorylation and SG inhibition by ZIKV-NS2B^V35A^ recombinant virus in Vero cells. **(A)** Western blot analysis of Vero cells mock-infected, infected with ZIKV-NS2B^WT^, and ZIKV-NS2B^V35A^ viruses, followed by treatment without or with As. **(B and C)** Immunostaining (B) and quantification of SGs (C) in Vero cells infected with ZIKV-NS2B^WT^ and ZIKV-NS2B^V35A^, followed by As treatment. **(D and E)** Immunostaining (D) and quantification of SGs (E) in Vero-PP1α^KO^ mix cells infected with ZIKV-NS2B^WT^ and ZIKV-NS2B^V35A^, followed by As treatment. Cells marked with "+" indicate ZIKV-infected cells that reduced SG formation, while cells marked with both "+" and yellow arrowheads indicate ZIKV-infected cells that failed to inhibit SG formation. The white scale bar represents 10μm. Error bars represent the standard deviation of three independent experiments, with a total of 150 cells counted in each experiment. Statistical significance was determined using Student’s t-test, where n.s. denotes no statistical significance, *P < 0.05, **P < 0.01, and ***P < 0.001.
(TIF)

S7 Fig ZIKV-NS2B^V35A^ exhibited reduced apoptosis levels. **(A)** Immunostaining of organoids exposed to ZIKV-NS2B^WT^, ZIKV-NS2B^V35A^, or mock treatment (scale bars, 100 μm).
(TIF)

S1 Data Excel includes values used to generate graphs. (XLSX)
