# Supplementary figures and images for: The NS2B-PP1α-eIF2α axis: Inhibiting stress granule formation and Boosting Zika virus replication
Source: PLoS Pathog. 2024 Jun 27;20(6):e1012355. doi: 10.1371/journal.ppat.1012355 (PMC11236161; doi:10.1371/journal.ppat.1012355)

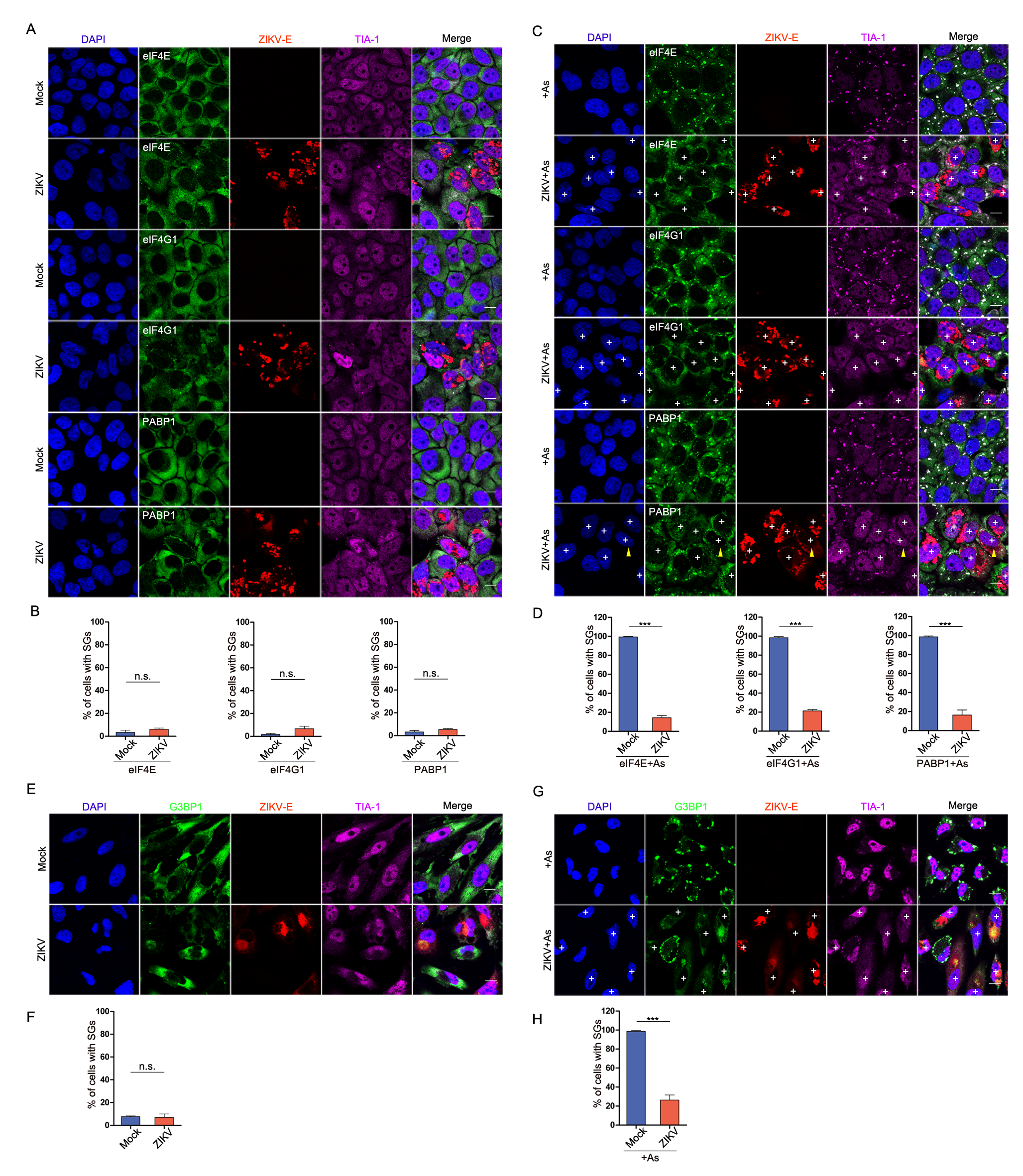

Supplement: S1 Fig — (A) Immunofluorescence analysis was performed to visualize protein aggregation in Hela cells that were either mock-infected or infected with ZIKV (MOI of 0.3) for 36 hours. (B) The percentage of cells containing SGs was quantified in three independent experiments based on the observations in panel (A). (C) Hela cells were mock-infected or infected with ZIKV (MOI of 0.3) for 36 hours, followed by treatment with As (200uM,1h). (D) The percentage of cells containing stress granules was quantified in three independent experiments based on the observations in panel (C). (E) A549 cells were mock-infected or infected with ZIKV (MOI of 0.1) for 24 hours, and then subjected to immunofluorescence staining to visualize G3BP1 (green) and ZIKV-E (red). (F) The percentage of cells containing stress granules was quantified in three independent experiments based on the observations in panel (E). (G) A549 cells were mock-infected or infected with ZIKV (MOI of 0.1) for 24 hours, followed by treatment with As (500uM,1h). (H) The percentage of cells containing stress granules was quantified in three independent experiments based on the observations in panel (G). Cells labeled with "+" indicate ZIKV-infected cells that inhibited SG formation, while cells marked with both "+" and yellow arrowheads indicate those that did not. The white scale bar represents a length of 10 μm. Data is presented as mean ± SD (n = 3), with a total of 150 cells counted in each experiment. Statistical analysis was conducted using Student’s t-test, where n.s. indicates no significant difference, and *P < 0.05, **P < 0.01, ***P < 0.001 indicate significant differences. (TIF) [file ppat.1012355.s001.tif]

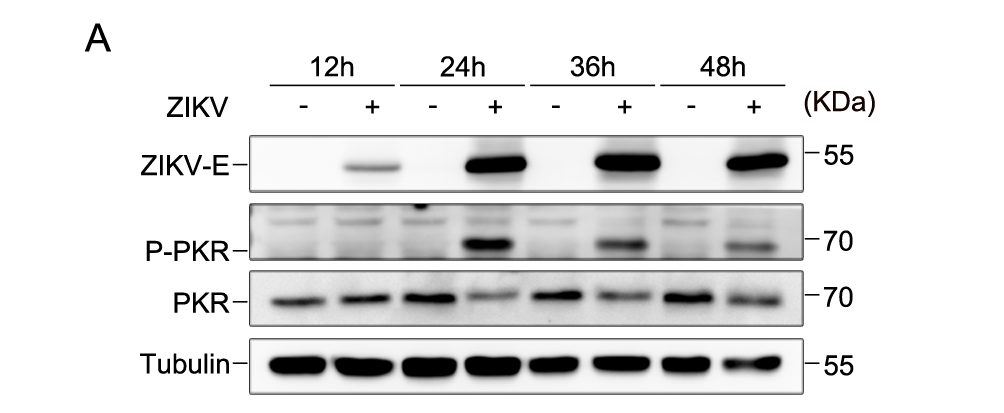

Supplement: S2 Fig — (A) Western blot analysis depicting the activation state of PKR during ZIKV infection. (TIF) [file ppat.1012355.s002.tif]

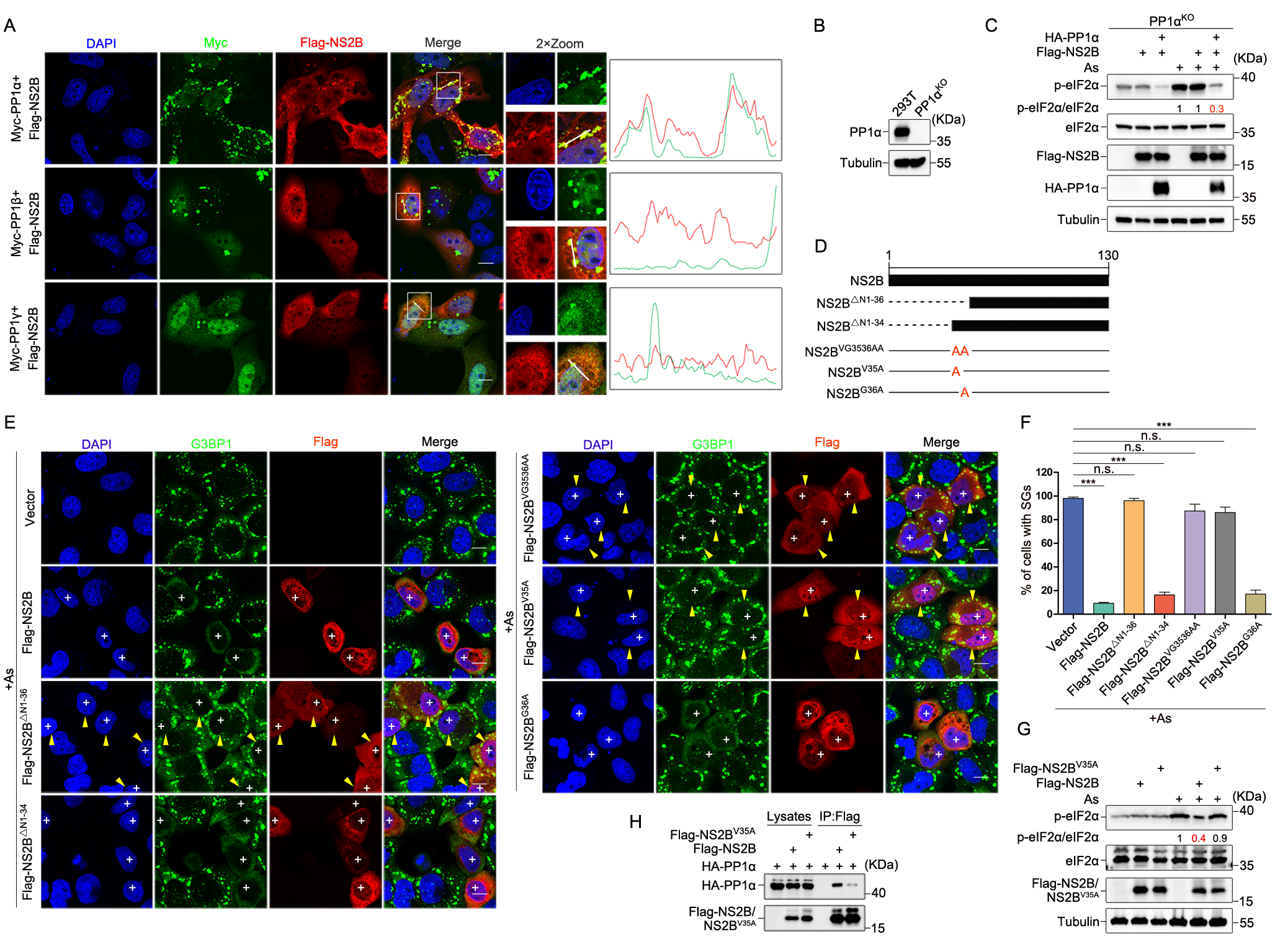

Supplement: S3 Fig — (A) Fluorescence intensity profiles of Myc-PP1α, Myc-PP1β, Myc-PP1γ, and Flag-NS2B in Hela cells transfected with Myc-PP1α, Myc-PP1β, Myc-PP1γ, and Flag-NS2B for 24 hours. (B) Western blot analysis comparing PP1α protein expression levels between HEK293T WT cells and HEK293T-PP1αKO cell clones. (C) Western blot analysis of HEK293T-PP1αKO cells transfected with empty vector, Flag-NS2B, or Flag-NS2B and HA-PP1α with synonymous mutations, followed by treatment with or without As. (D) Schematic diagrams of N-terminally truncated or point mutants of NS2B. (E and F) Immunostaining (E) and quantification of SGs (F) of N-terminally truncated or point mutants of NS2B. (G) Western blot analysis of HEK293T cells transfected with empty vector, Flag-NS2B, or Flag-NS2BV35A, followed by treatment with or without As. (H) Immunoprecipitation assays comparing the interaction of NS2B, NS2BV35A, and HA-PP1α in HEK293T cells. The symbol "+" denotes cells expressing NS2B or its mutants that inhibited SG formation, while cells with both "+" and yellow arrowheads indicate cells expressing NS2B or its mutants that failed to inhibit SG formation. The white scale bar represents a length of 10 μm. The data is presented as mean ± SD (n = 3). In each experiment, 150 cells were counted. Statistical analysis was conducted using Student’s t-test, with n.s. indicating non-significant results, and *P < 0.05, **P < 0.01, ***P < 0.001 denoting significant differences. (TIF) [file ppat.1012355.s003.tif]

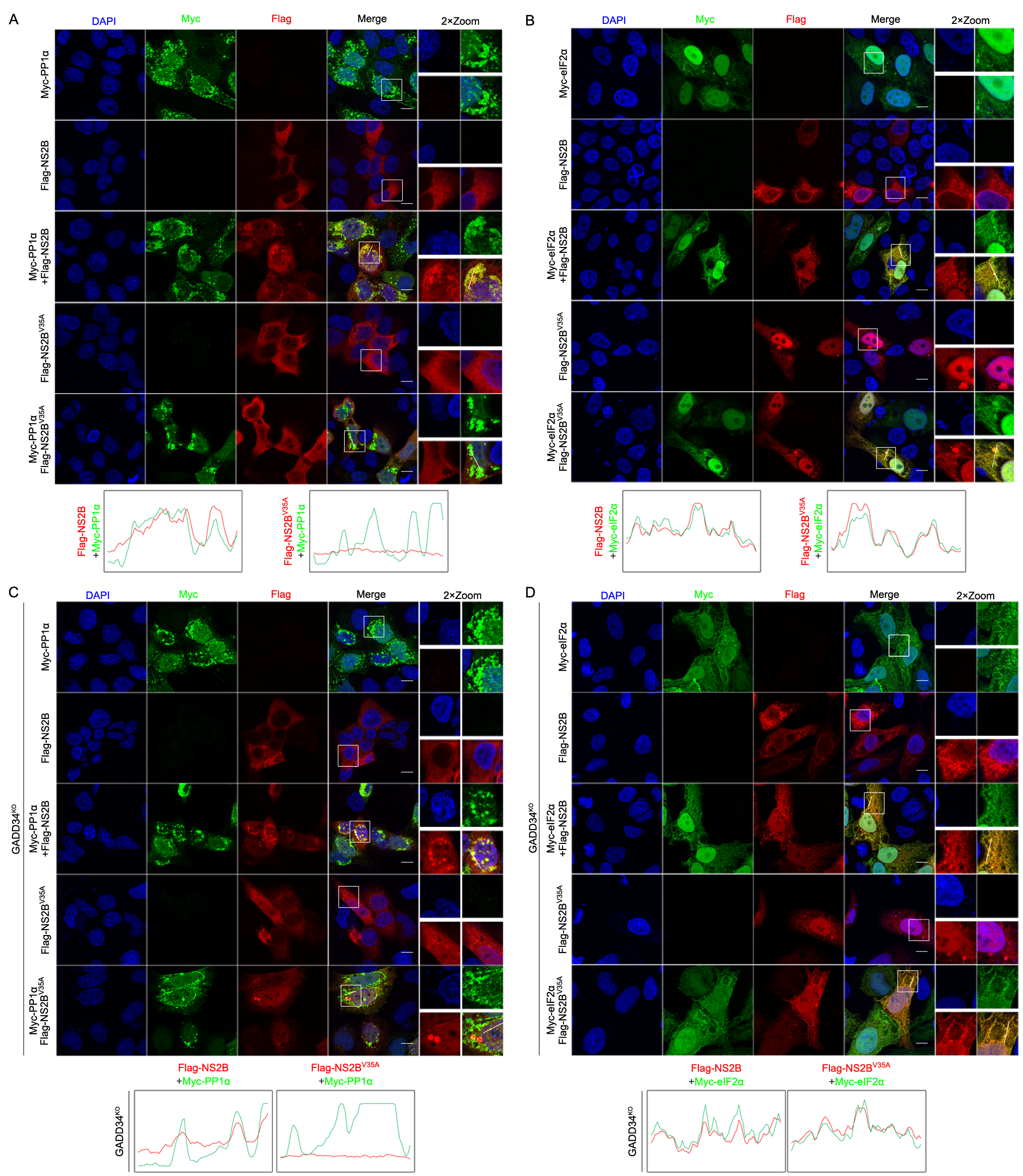

Supplement: S4 Fig — (A and C) Hela cells (A) and Hela-GADD34KO cells (C) were transfected with Myc-PP1α, Flag-NS2B, Myc-PP1α and Flag-NS2B, Flag-NS2BV35A, Myc-PP1α and Flag-NS2BV35A plasmids for 24 hours. The cells were subsequently immunostained with anti-Myc (green) and anti-Flag (red) antibodies, and the fluorescence intensity profile of PP1α (green) and NS2B, NS2BV35A (red) was measured. A white scale bar of 10 μm was included for reference. (B and D) Hela cells (B) and Hela-GADD34KO cells (D) were transfected with Myc-eIF2α, Flag-NS2B, Myc-eIF2α and Flag-NS2B, Flag-NS2BV35A, Myc-eIF2α and Flag-NS2BV35A plasmids for 24 hours. The cells were immunostained with anti-Myc (green) and anti-Flag (red) antibodies, and the fluorescence intensity profile of eIF2α (green) and NS2B, NS2BV35A (red) was measured. A white scale bar of 10 μm was included for reference. (TIF) [file ppat.1012355.s004.tif]

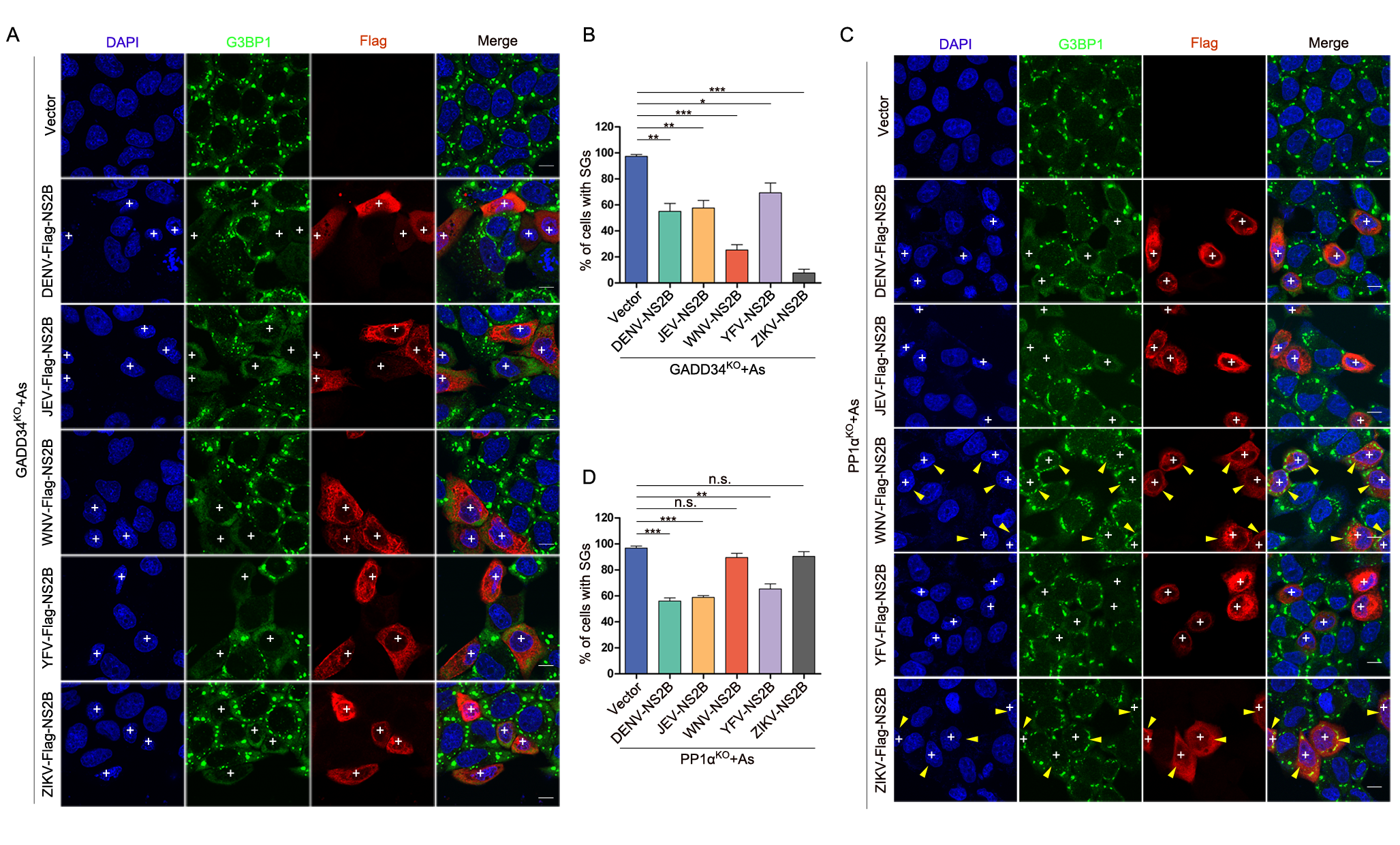

Supplement: S5 Fig — (A and B) Investigation of Flavivirus NS2B’s influence on SG formation in Hela-GADD34KO cells. Cells were transfected with either empty vector or plasmids encoding DENV-Flag-NS2B, JEV-Flag-NS2B, WNV-Flag-NS2B, YFV-Flag-NS2B and ZIKV-Flag-NS2B for 24 hours, followed by a 1-hour treatment with As before harvesting. (A) Immunostaining with anti-G3BP1 (green) and anti-Flag (red) antibodies; (B) Analysis of the percentage of cells containing SGs from the experiments in panel (A). (C and D) Hela-PP1αKO cells were transfected with empty vector, DENV-Flag-NS2B, JEV-Flag-NS2B, WNV-Flag-NS2B, YFV-Flag-NS2B and ZIKV-Flag-NS2B plasmids for 24 hours and subsequently treated with As for 1 hour before harvesting; (C) Immunostaining with anti-G3BP1 (green) and anti-Flag (red); (D) Analysis of the percentage of cells containing SGs from the experiments in panel (C) Cells with the symbol “+” represent cells expressing NS2B that effectively inhibit SG formation, while cells marked with both “+” and yellow arrowheads indicate NS2B-expressing cells that fail to inhibit SG formation. The white scale bar indicates 10 μm. Error bars denote the standard deviation of results from three independent experiments, with 150 cells counted in each experiment. Statistical significance was determined using Student’s t-test, where n.s. indicates no significance, *P < 0.05, **P < 0.01, and ***P < 0.001. (TIF) [file ppat.1012355.s005.tif]

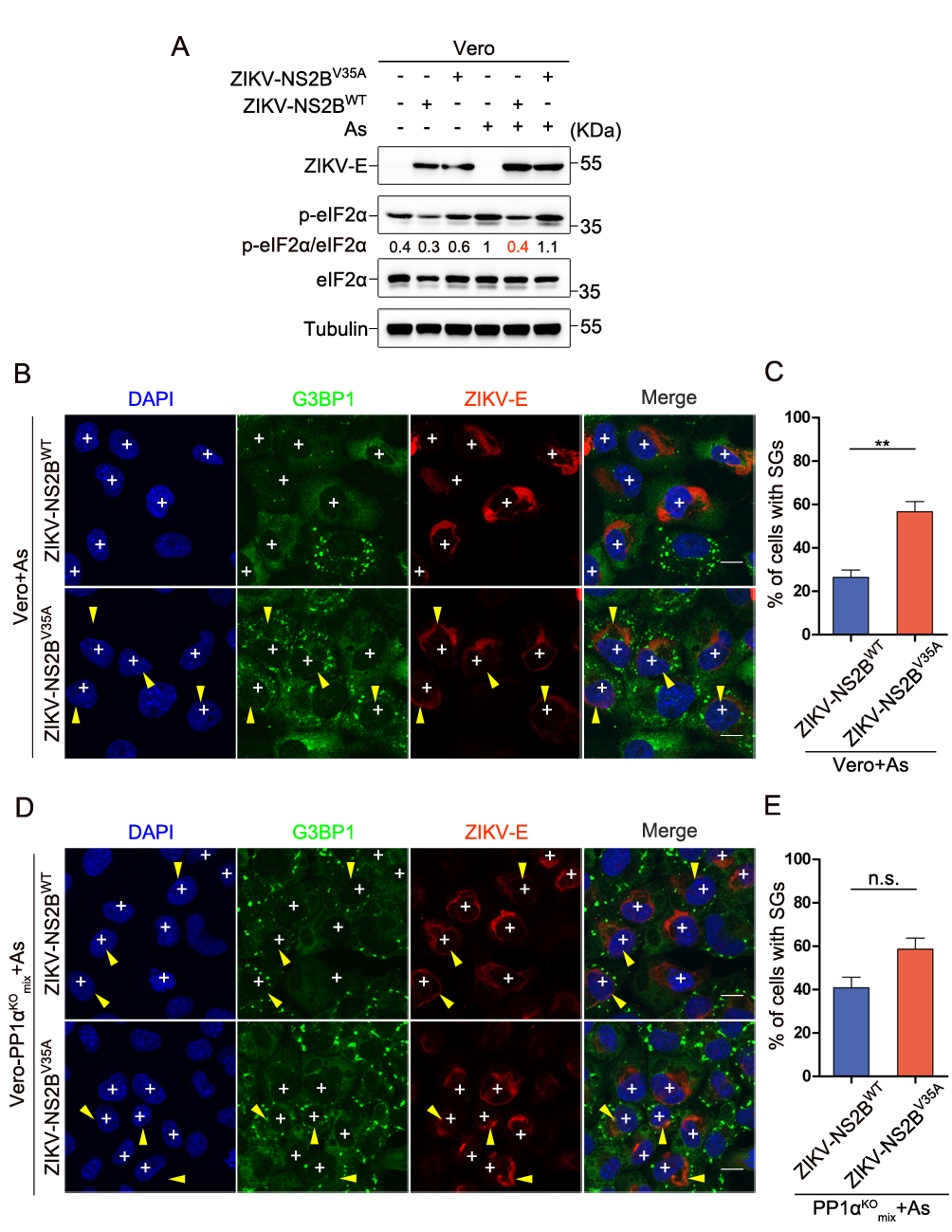

Supplement: S6 Fig — (A) Western blot analysis of Vero cells mock-infected, infected with ZIKV-NS2BWT, and ZIKV-NS2BV35A viruses, followed by treatment without or with As. (B and C) Immunostaining (B) and quantification of SGs (C) in Vero cells infected with ZIKV-NS2BWT and ZIKV-NS2BV35A, followed by As treatment. (D and E) Immunostaining (D) and quantification of SGs (E) in Vero-PP1αKO mix cells infected with ZIKV-NS2BWT and ZIKV-NS2BV35A, followed by As treatment. Cells marked with "+" indicate ZIKV-infected cells that reduced SG formation, while cells marked with both "+" and yellow arrowheads indicate ZIKV-infected cells that failed to inhibit SG formation. The white scale bar represents 10μm. Error bars represent the standard deviation of three independent experiments, with a total of 150 cells counted in each experiment. Statistical significance was determined using Student’s t-test, where n.s. denotes no statistical significance, *P < 0.05, **P < 0.01, and ***P < 0.001. (TIF) [file ppat.1012355.s006.tif]

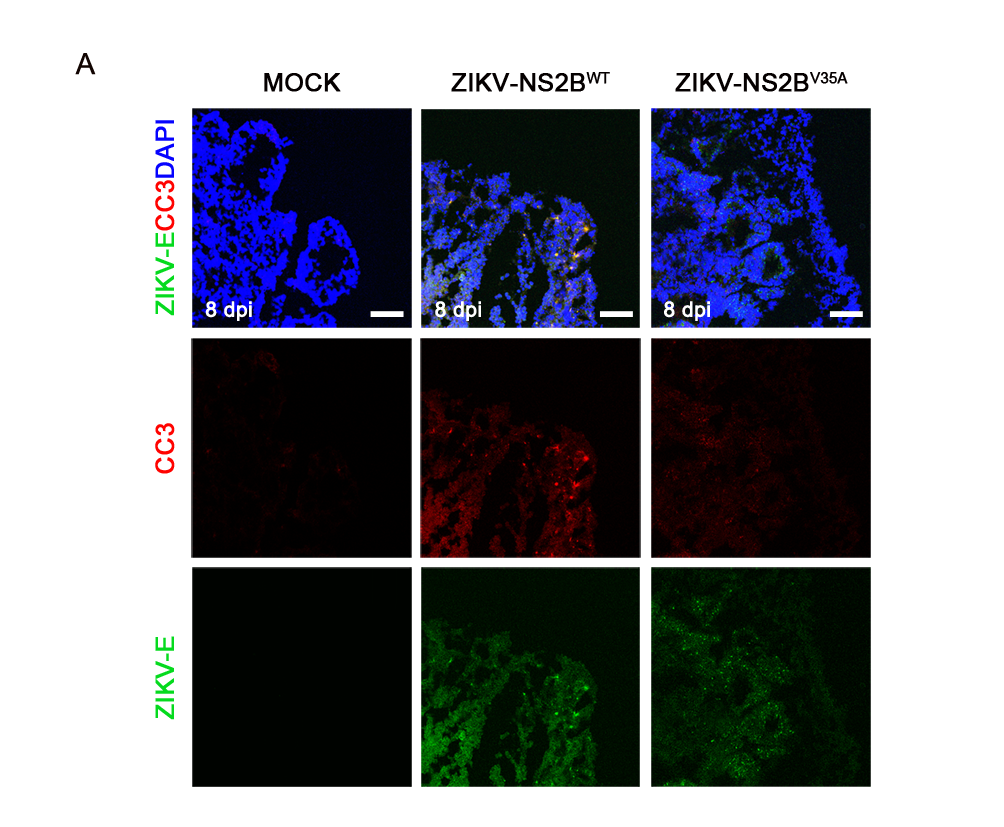

Supplement: S7 Fig — (A) Immunostaining of organoids exposed to ZIKV-NS2BWT, ZIKV-NS2BV35A, or mock treatment (scale bars, 100 μm). (TIF) [file ppat.1012355.s007.tif]
